# From armadillos to sloths: Patterns and variations in xenarthran coronary anatomy

**DOI:** 10.1002/ar.70073

**Published:** 2025-10-22

**Authors:** Wilson Viotto‐Souza, André Luiz Quagliatto Santos, Marcelo Abidu‐Figueiredo, Thaís Aparecida Silva, Rodrigo Ananias Barreiros Silva, Paulo de Souza‐Junior

**Affiliations:** ^1^ Universidade Federal de Uberlândia (UFU) Uberlândia Brazil; ^2^ Fundação Presidente Antônio Carlos (UNIPAC) Uberlândia Brazil; ^3^ Universidade Federal Rural do Rio de Janeiro (UFRRJ) Seropédica Brazil; ^4^ Universidade Federal do Pampa (UNIPAMPA) Uruguaiana Brazil

**Keywords:** cardiovascular system, comparative anatomy, coronary dominance, neotropical mammals, wildlife anatomy

## Abstract

Species of the superorder *Xenarthra* play a vital ecological role in the Neotropics. Despite their evolutionary significance, anatomical studies on their coronary circulation remain scarce. This study investigated the coronary anatomy of 82 hearts from nine *Xenarthra* species across the *Dasypodidae*, *Myrmecophagidae*, and *Bradypodidae*. The right coronary artery (RCA) originated from the right aortic sinus in all specimens and typically emitted atrial, ventricular, and marginal branches, as well as the subsinuosal interventricular branch (94.9%). A common trunk of the left coronary artery (LCA) was identified in 89% of hearts, typically divided into paraconal interventricular and circumflex branches; when absent, these branches arose directly from the aortic sinus. Vessels coursed predominantly epicardially (74.4%), with intramyocardial paths and myocardial bridges restricted to PIVB. Anastomoses between interventricular branches were present in 28.6% of cases. Coronary dominance was predominantly balanced (92.3%), although classifications varied across the three criteria applied, underscoring method dependence. Quantitative comparisons revealed significant interspecific variation in the number and distribution of ventricular branches, with *Cabassous tatouay* exhibiting the highest mean from the PIVB. Family‐level patterns and ecological associations were observed, including anastomoses restricted to Myrmecophagidae, simplified branching in sloths consistent with low metabolic rates, and greater redundancy with intramyocardial courses in fossorial or foraging taxa. This study advances the understanding of xenarthran coronary anatomy and provides a comparative framework for evolutionary interpretation and veterinary care of these often‐threatened mammals.

## 1 INTRODUCTION

The superorder Xenarthra (class Mammalia, subclass Theria, and infraclass Eutheria) is represented by 32 extant species distributed across four families: Dasypodidae (armadillos), with 21 species; Myrmecophagidae (anteaters), with four species; Bradypodidae (three‐toed sloths), with five species; and Megalonychidae (two‐toed sloths), with two species (Madsen et al., 2001; Miranda et al., 2023; Murphy et al., 2001; Springer et al., 2003). These four families are grouped into two orders: Pilosa, which includes two suborders, Folivora, represented by sloths, and Vermilingua, represented by anteaters, and Cingulata, represented by armadillos (Delsuc et al., 2001; Glass, 1985; McKenna & Bell, 1997; Murphy et al., 2001; Springer et al., 2003; Vizcaíno & Scillato‐Yané, 1995).

Xenarthrans are among the few animals that have diversified in a geographically restricted area, representing a successful evolutionary tree among South American mammals (Engelmann, 1985; Pough et al., 1993). The monophyletic clade of placental mammals that exhibits numerous primitive synapomorphic traits is the xenarthrans, suggesting the hypothesis that they are the most primitive living common ancestor of the Eutheria recorded in the Southern Hemisphere (Delsuc et al., 2001; Engelmann, 1985; Murphy et al., 2001). However, anthropogenic pressures contribute to the classification of species such as *Priodontes maximus* (giant armadillo), *Bradypus torquatus* (collared sloth), and *Myrmecophaga tridactyla* (giant anteater) as vulnerable to extinction (Anacleto et al., 2014; Chiarello et al., 2022; Miranda et al., 2014).

The heart's blood supply is provided by the coronary arteries, where 5 to 15% of the cardiac output is allocated for cardiovascular function in domestic mammals. These arteries emit branches that may be entirely superficial (epicardial course), deep (intramyocardial course), or mixed (course with myocardial bridges) (Engen, 2006; Getty, 1986; Guyton & Hall, 1997; Loukas et al., 2006; Rai et al., 2020; Singh, 2018).

Since the pioneering studies of Banchi (1904), there has been a continued interest in identifying which artery plays a dominant role in irrigating myocardial tissue, which can be classified into three categories: left, right, or balanced, as described by Schummer et al. (1981). Several methods have been employed to determine coronary dominance, including investigating which artery gives rise to the subsinuosal interventricular branch, which one extends beyond the apex of the heart, and analyzing the length and relative number of branches emanating from the coronary arteries (Scansen, 2017; Viotto‐Souza et al., 2023).

The organization of coronary circulation in mammals can be better understood within an evolutionary framework, tracing back to older lineages such as reptiles, marsupials, and monotremes. Reptiles, representing a pre‐mammalian state, exhibit a coronary arterial supply that reflects the duality of their myocardium, composed of an inner spongy layer and an outer compact layer, with considerable variation in coronary vessel configuration (Jensen et al., 2014; MacKinnon & Heatwole, 1981). In contrast, monotremes already display a more mammal‐like arterial arrangement, with two main coronary arteries (right and left) running epicardially on the heart surface, although their venous return still lacks a true coronary sinus, resembling the avian condition (Dowd, 1969). Marsupials, however, deviate from this pattern: in the possum Trichosurus vulpecula, coronary arteries penetrate deeply into the myocardium, resulting in vessels that are not visible epicardially, a strikingly intramyocardial distribution distinct from most eutherians (Dowd, 1974). By situating the coronary circulation of xenarthrans in relation to these older lineages, it is possible to evaluate whether their anatomy aligns more closely with the epicardial arrangement of monotremes or with the intramyocardial variant of marsupials, thereby refining our understanding of cardiovascular adaptations within this superorder.

The functional importance of coronary circulation has driven anatomical investigations into the coronary arteries and their main branches in domestic mammals, such as *Bos taurus* (Correia‐Oliveira et al., 2013; Correia‐Oliveira, Moraes, et al., 2014), *Canis lupus familiaris* (Biasi et al., 2013; Büll & Martins, 2002; Donald & Essex, 1954; Oliveira et al., 2010), *Felis catus* (Biasi et al., 2012; Borelli, 2014; Monfared et al., 2013; Schiller, 1957), *Mus musculus* (Yoldas et al., 2010), *Oryctolagus cuniculus domesticus* (Bahar et al., 2007; Correia‐Oliveira, Oliveira, et al., 2014), *Sus scrofa domesticus* (Moura Junior et al., 2008); and in wild mammals such as *Camelus bactrianus* (Yuan et al., 2009), *Mazama gouazoubira* (Zaniboni et al., 2021), *Panthera leo* (Schiller, 1957), *Puma concolor* (Viotto‐Souza et al., 2017), *Sapajus apella* (Rade et al., 2006), *Sus scrofa* (Ribeiro et al., 2014), and *Cuniculus paca* (Ávila et al., 2009). In xenarthrans, descriptive records of coronary circulation have been found in anteaters (*Myrmecophaga tridactyla* and *Tamandua tetradactyla*), showing balanced, left, and right types of irrigation (Cruvinel et al., 2019; Pinheiro et al., 2014; Santos et al., 2020).

The accuracy of anatomical investigations regarding the circulatory system underpins veterinary procedures in conservation units and zoos, as well as aiding in the elucidation of evolutionary inferences. Although preliminary descriptions of the heart in xenarthran species are available, literature on coronary circulation is scarce. Motivated by the essential role of xenarthrans in ecosystems, the anatomical and physiological importance of the heart, and the lack of descriptions for these species, this study aimed to investigate coronary circulation in the hearts of the superorder Xenarthra.

## 2 MATERIALS AND METHODS

### 2.1 Sample collection

A total of 82 hearts from carcasses were analyzed, with most of the specimens collected from animals found dead on highways in the states of Minas Gerais (MG) and Rio de Janeiro (RJ), Brazil. Some carcasses were collected from the Veterinary Hospital of the Universidade Federal de Uberlândia, resulting from natural death or disease. The collection of these animals was conducted under the cooperation agreement no. 002/2011 between Universidade Federal de Uberlândia and IBAMA, with authorization from SISBIO, no. 49266‐1. As previously mentioned, all specimens died naturally or from disease, and no experiments were conducted on living animals. Consequently, under Brazilian legislation (Law No. 11,794 of 2008), this study is exempt from requiring approval from the Ethics Committee on Animal Use (CEUA). The hearts of these specimens represent nine species of the superorder Xenarthra from two of Brazil's main biomes: the Cerrado and the Atlantic Forest (Table 1).

**TABLE 1:** Anatomical sampling of hearts from species of the superorder Xenarthra (*n* = 82) for studying coronary irrigation.

Superorder	Order	Family	Species	Adult			Juvenile			Total	Location
				♂	♀	?	♂	♀	?		
Xenarthra	Cingulata (*n* = 15)	Dasypodidae (*n* = 15)	*Cabassous uncintus*	2	‐	‐	‐	‐	‐	2	MG
			*Cabassous tatouay*	2	1	‐	‐	‐	‐	3	MG
			*Dasypus novemcinctus*	1	‐	2	1	‐	‐	4	MG
			*Euphractus sexcinctus*	2	1	‐	‐	‐	‐	3	MG
			*Tolypeutes tricinctus*	2	1	‐	‐	‐	‐	3	MG
	Pilosa (*n* = 67)	Myrmecophagidae (*n* = 61)	*Myrmecophaga tridactyla*	17	17	5	6	3	‐	48	MG
			*Tamandua tetradactyla*	7	6	‐	‐	‐	‐	13	MG
		Bradypodidae (*n* = 6)	*Bradypus variegatus*	1	1	‐	‐	‐	‐	2	MG
			*Bradypus crinitus*	2	2	‐	‐	‐	‐	4	RJ
Total				36	29	7	7	3	0	82	

Abbreviations: MG, State of Minas Gerais; RJ, State of Rio de Janeiro.

The taxonomic identification of the species was carried out using the collections database, based on the phenotypic description presented in the literature. To determine the sex (male or female), whenever possible, morphological identification of the genital organs was performed, taking into account the integrity of the organs. Regarding age classification (juveniles or adults), the criterion based on observed feeding behavior was adopted, classifying lactating animals (approximately up to 9 months of age) as juveniles and the others as adults (Maia, 2002; Medri et al., 2011).

### 2.2 Anatomical preparation of xenarthran hearts using the dissection technique

After species and sex identification (whenever possible), the cadaver was positioned in dorsal recumbency to facilitate the sternotomy procedure (from the xiphoid process to the manubrium) using rib shears, allowing access to the thoracic cavity with full visualization of the heart. Once the heart was accurately located, the great vessels at the base, along with the esophagus, trachea, and nerves, were sectioned, ensuring a wide margin of distance from the heart to avoid its perforation. Following the complete removal of the heart from the thoracic cavity, adjacent tissues were carefully dissected and removed to preserve the heart, the great vessels, and the pericardial sac. Dissection continued with the complete removal of the pericardial sac, and the heart was washed under running water while being gently massaged to dilute and remove as many clots as possible from the coronary arteries.

With the heart properly isolated, the great vessels at the base were sectioned as close as possible to their origin to facilitate future handling of the study material. This allowed visualization of the coronary ostia, conal ostium, accessory ostium, and other branches that may arise from the aortic sinus. After individually identifying each ostium, the respective vessels were cannulated with catheters (according to the vessels' caliber) for the injection of a latex solution mixed with primary color dyes: blue, yellow, or red. The vessels were individually stained as follows: the right coronary artery in blue, the left coronary artery in yellow, the conal artery in red, the paraconal interventricular branch in yellow (when originating directly from the aortic ostium), and the circumflex branch in yellow (when originating directly from the aortic ostium). The accessory coronary artery was not stained due to its small diameter. Each vessel injected with the aforementioned solution was ligated at its origin using 3–0 blue polypropylene suture thread. The hearts were stored in 500 mL containers with a 10% formaldehyde solution for fixation.

After 15 days of fixation, the coronary arteries and their branches were dissected using forceps and an articulated magnifying glass (10× magnifier, BCMED®). Macrophotographs (Nikon Coolpix® L820 camera, 16MP) were taken to illustrate the distribution of the coronary arteries and their branches. The nomenclature used in this study followed the *Nomina Anatomica Veterinaria* (International Committee on Veterinary Gross Anatomical Nomenclature [ICVGAN], 2017).

### 2.3 Identification and counting of coronary arteries and their branches

The presence or absence of vessels originating from the aortic bulb sinus was identified, including the right coronary artery (RCA) and left coronary artery (LCA), the conal artery (CA), the accessory coronary artery (ACA), the paraconal interventricular branch (PIVB), and the circumflex branch (CB). Additionally, the site of origin, presence, and absence of the left and right marginal branches (LMB and RMB), as well as the origin and presence or absence of the septal branch (SB), were recorded. The origin, presence, and absence of the paraconal interventricular branch (PIVB) and the subsinuous interventricular branch (SIVB) were also identified, along with the level reached (pre‐apical, apical, or post‐apical) by the PIVB and SIVB. The number of ventricular (vbRV—ventricular branches for Right Ventricle and vbLV—ventricular branches for Left Ventricle) and atrial (abRA—atrial branches for Right Atrium and abLA—atrial branches for Left Atrium) branches originating from the RIVP, RIVS, RC, AC, and ACD was counted. Data were tabulated using GraphPad Prism 8.0.2®.

### 2.4 Identification and counting of the origin and distribution of coronary irrigation

The presence of coronary ostia for heart irrigation was identified in the aortic bulb sinus, originating the following vessels: LCA, RCA, PIVB, CB, CA, and ACA. Following the identification of these vessels, the number of ostia for each side of the aortic bulb sinus (left or right) was counted.

The distribution of coronary irrigation presented three patterns: epicardial route (vessels on the myocardial surface), intramyocardial route (vessels that penetrate the myocardium without resurfacing on the heart's surface), and mixed route (epicardial or intramyocardial route, with the presence of myocardial bridges over a specific section of a vessel). The presence or absence of myocardial bridges was also counted, and their location was identified.

The presence of communications between thin ventricular vessels, known as anastomoses, was identified. These anastomoses were recorded based on their location relative to the heart (basal or apical) and their relation to potential coronary vessels (PIVB, CB, RCA, SIVB, LMB, RMB) from which the anastomosed ventricular branches originated.

### 2.5 Classification of the coronary irrigation pattern

The determination of the coronary irrigation dominance pattern (left, right, or balanced) was established based on three criteria: the first considered dominance according to the origin of the interventricular branches (PIVB and SIVB), directly or indirectly, from the aortic bulb sinuses (left, right, or both); the second considered the total number of ventricular branches directly originating from each aortic bulb sinus (left or right), with predominant blood flow being left, right, or balanced; and the third considered the total number of ventricular branches destined for each ventricle (right or left).

### 2.6 Statistical analysis

The quantitative descriptive statistics displayed the arithmetic mean and standard deviation regarding the number of ventricular branches for each species. The comparison between the specimens from the Bradypodidae, Dasypodidae, and Myrmecophagidae families evaluated the following variables: total number of ventricular and atrial branches; number of ventricular branches for each ventricle; and the number of ventricular branches from each interventricular branch. One‐way analysis of variance (ANOVA) was performed, complemented by the Tukey test and Student's *t* test.

For the comparison of the mean number of ventricular branches between age and sex, the mean total number of ventricular branches originating from the aortic bulb sinuses (left and right) was used, and the Student's *t* test (independent samples) was applied.

In all tests, a significant level of *p* < 0.05 was considered. All statistical analyses were performed using the GraphPad Prism 8.0.2® software.

## 3 RESULTS

### 3.1 Descriptive angioarchitecture of the coronary arteries and their branches

**FIGURE 1:**
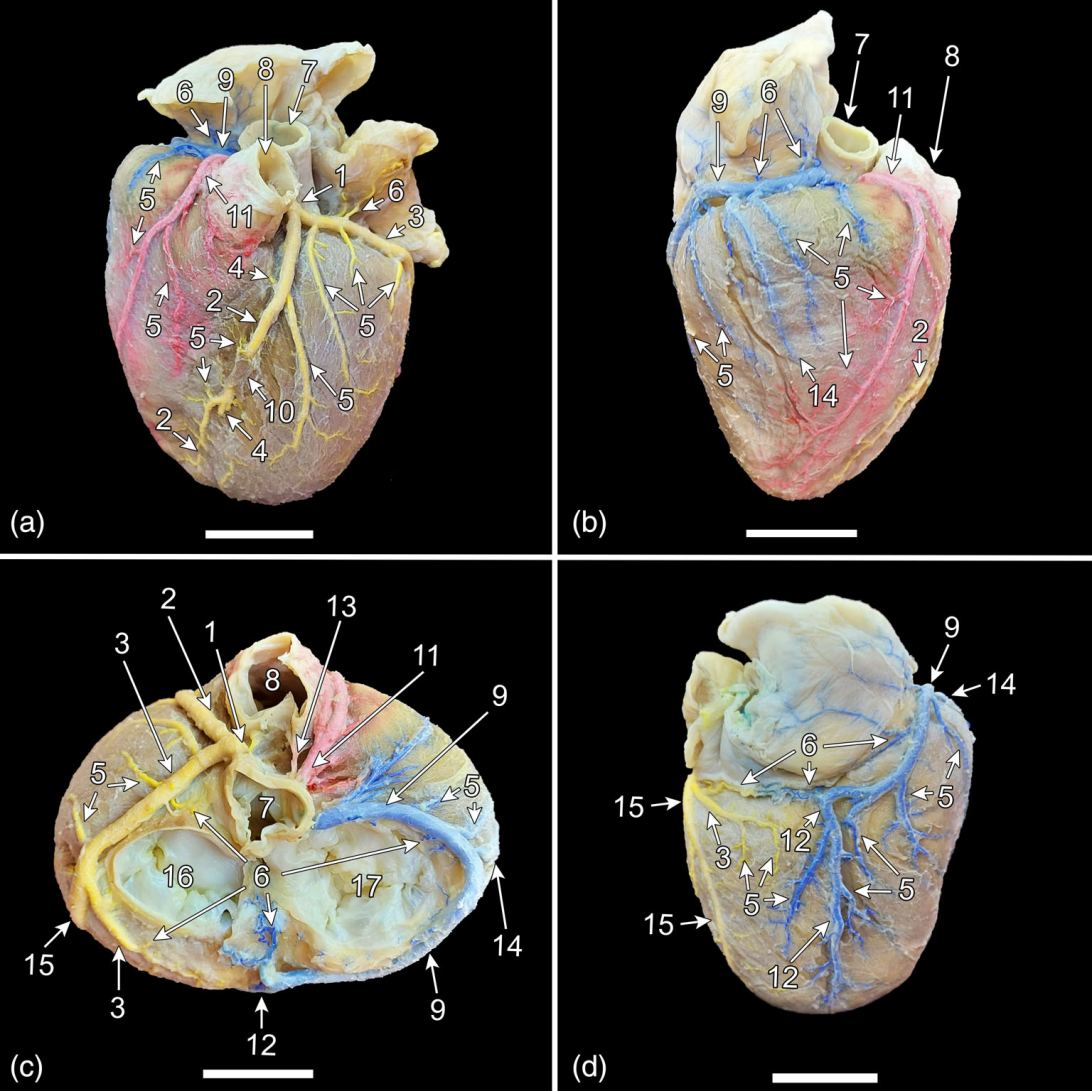
Macrophotography of the heart of an adult female *Myrmecophaga tridactyla*. View of the left (a) and right (b) sides of the heart; dorsal view of the heart (base) after the removal of the atria (c); view of the transition between the left and right sides of the heart (d). Left coronary artery (1); paraconal interventricular branch (2); circumflex branch (3); septal branch (4); ventricular branches (5); atrial branches (6); aortic artery (7); pulmonary trunk (8); right coronary artery (9); myocardial bridge (10); conal artery (11); subsinuosal interventricular branch (12); accessory coronary artery (13); right marginal branch (14); left marginal branch (15); left atrioventricular ostium (16); right atrioventricular ostium (17). Scale bar: 2 cm.

**FIGURE 2:**
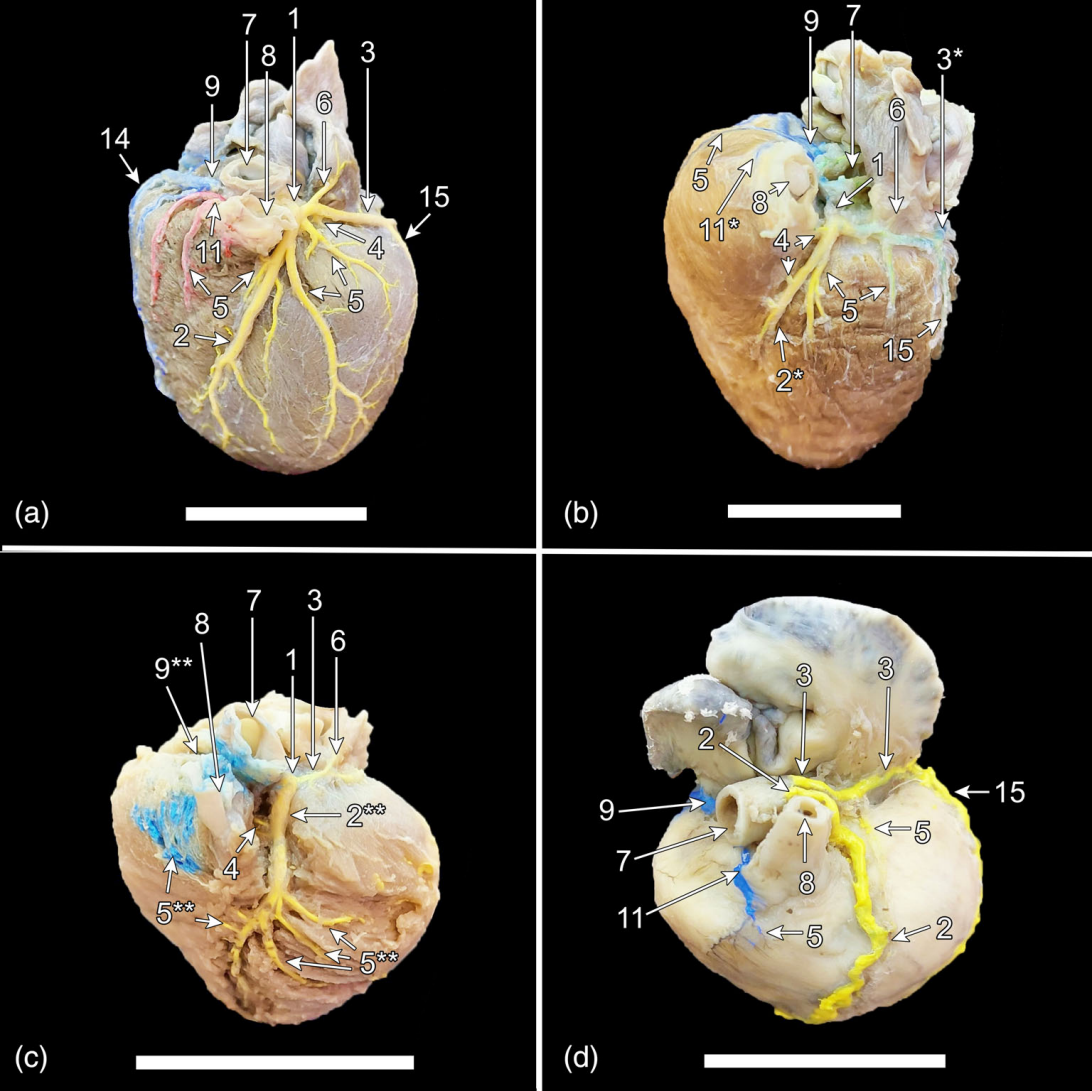
Macrophotography of the left side of Xenarthra hearts. Heart of a male juvenile *Myrmecophaga tridactyla* (a); heart of a male adult *Tamandua tetradactyla* (b); heart of a male adult *Euphractus sexcinctus* (c); heart of a female adult *Bradypus variegatus* (d). Left coronary artery (1); paraconal interventricular branch (2); paraconal interventricular branch with partial intramyocardial course (2); paraconal interventricular branch with total intramyocardial course (2*); circumflex branch (3); septal branch (4); ventricular branches (5); ventricular branches with total intramyocardial course (5**); atrial branches (6); aortic artery (7); pulmonary trunk (8); right coronary artery (9); right coronary artery with total intramyocardial course (9**); conal artery (11); conal artery originating from the right coronary artery (11*); right marginal branch (14); left marginal branch (15). Scale bar: 2 cm.

**FIGURE 3:**
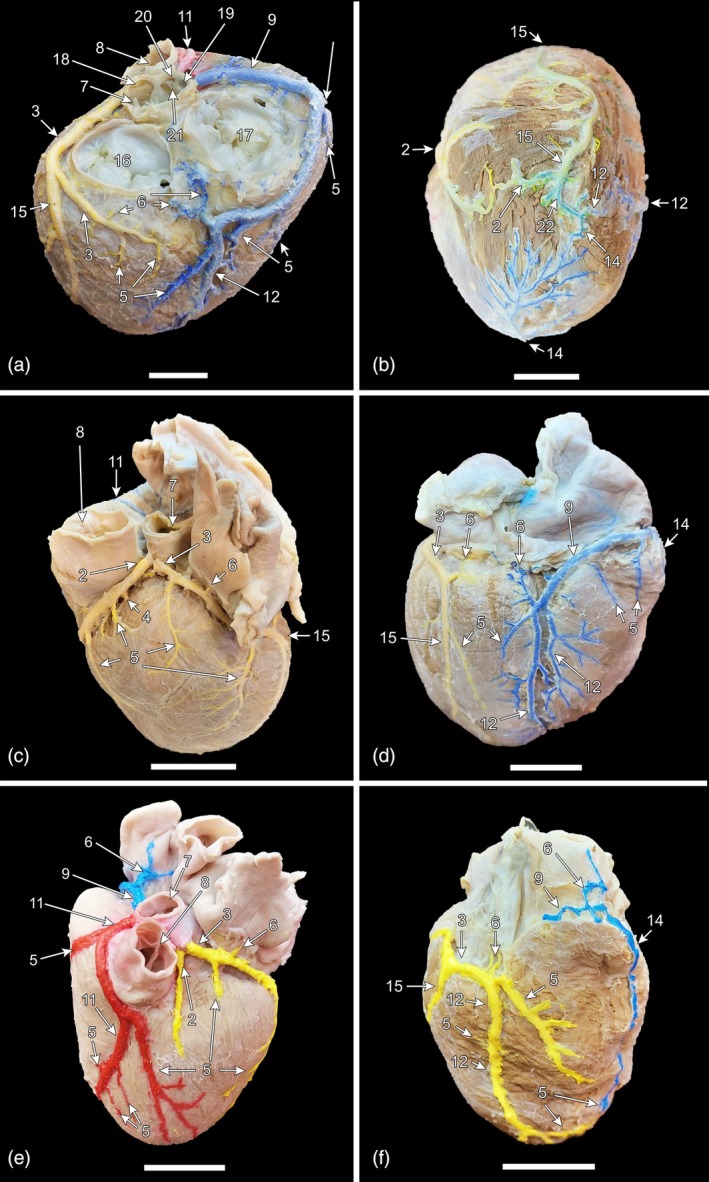
Macrophotography of *Myrmecophaga tridactyla* hearts. Dorsal view of the heart (base) of an adult female for visualization of the ostia (a). Ventral view of the heart (apex) of an adult female for visualization of anastomoses (b); view of the left side of the heart of an adult male for visualization in the region of the left sinus of the bulbus aorticus, the individualized origin of the paraconal interventricular branch and the circumflex branch with the absence of the left coronary artery (c). View of the transition between the left and right sides of the heart of an adult male for visualization of the presence of two subsinuous interventricular branches running their path along the subsinuous interventricular groove (d). Left side view of the heart of an adult male for visualization of the conal artery's path, which originates from the right sinus of the bulbus aorticus and projects to the left side of the heart, following the course along the paraconal interventricular groove (e). View of the transition between the left and right sides of the heart of an unidentified‐sex specimen for visualization of the origin of the subsinuous interventricular branch that originates from the circumflex branch (f). Paraconal interventricular branch (2); circumflex branch (3); septal branch (4); ventricular branches (5); atrial branches (6); aortic artery (7); pulmonary trunk (8); right coronary artery (9); conal artery (11); subsinuous interventricular branch (12); right marginal branch (14); left marginal branch (15); left atrioventricular ostium (16); right atrioventricular ostium (17); left coronary artery ostium (18); right coronary artery ostium (19); conal artery ostium (20); accessory coronary artery ostium (21); anastomosis (22). Scale bar: 2 cm.

The presence of a common trunk of the left coronary artery (LCA) was consistently present in *B. crinitus*, *C. tatouay*, *D. novemcinctus*, *E. sexcinctus*, and *T. tricinctus* (100% of specimens). It was also frequent in *M. tridactyla* (91.7%) and *T. tetradactyla* (76.9%), but less regular in *B. variegatus* and *C. uncintus* (50% each). When present, the common trunk of the LCA arose from the left aortic sinus, followed a short course, and bifurcated into the paraconal interventricular branch (PIVB) and the circumflex branch (CB) (Figures 1, 2, 3).

The paraconal interventricular branch (PIVB) was present in 98.8% of the specimens. It usually originated from the LCA (90.1%), though in some cases (9.8%) it arose directly from the left aortic sinus. A duplicate PIVB occurred in one *T. tetradactyla*, while one *M. tridactyla* lacked this vessel. The PIVB typically followed the interventricular groove (97.6%) and was most often a single vessel. Its termination was predominantly apical (87.6%), whereas it ended pre‐apically in seven (8.6%) and post‐apically in three (3.7%) hearts.

The circumflex branch (CB) was present in 98.8% of the specimens, most commonly arising from the LCA (90.1%), though in a minority (9.8%), it originated directly from the left aortic sinus. A single *T. tetradactyla* lacked the CB. When present, the vessel coursed along the full length of the left coronary groove, reaching the subsinuous interventricular sulcus.

The septal branch (SB) most frequently originated from the PIVB (75.6%). This pattern predominated in *M. tridactyla*, *T. tetradactyla*, and Bradypodidae, while in a few cases it arose from the CB (three *M. tridactyla* and one *B. variegatus*). The SB was absent in some species, including *C. unicinctus*, *C. tatouay*, *E. sexcinctus*, and *T. tricinctus*.

The right coronary artery (RCA) was present in all specimens, consistently arising from the right aortic sinus and coursing along the coronary sulcus. Two additional vessels were also observed in parallel: the accessory coronary artery (ACA), a smaller‐caliber branch present in 14.6% of hearts (notably in *M. tridactyla*, *T. tetradactyla*, and *B. crinitus*), and the conal artery (CA), a larger vessel supplying the right ventricle. The CA originated directly from the right aortic sinus in 21.9% of specimens, mainly anteaters, while in the remaining 78% it arose from the RCA.

The subsinuous interventricular branch (SIVB) was present in 95.1% of the specimens, originating predominantly from the RCA (94.9%) and less often from the CB (5.1%). It was absent in four cases, restricted to *C. tatouay* and *E. sexcinctus*. Most hearts exhibited a single SIVB, though duplication occurred in one *M. tridactyla*. When present, the branch usually followed the interventricular sulcus (98.7%), with only one exception (*E. sexcinctus*). Termination was most often apical (60.2%), followed by post‐apical (34.6%) and, rarely, pre‐apical (5.1%).

The left marginal branch (LMB) was present in all specimens, consistently located along the left ventricular margin. It originated mainly from the CB (91.5%), but in some dasypodids (*C. tatouay* and *E. sexcinctus*) it arose from the PIVB, and in one *T. tetradactyla* from the RCA. Regarding its course, the LMB most frequently reached the apical level (68.3%), while in the remaining cases it ended pre‐apically. No post‐apical terminations were observed.

The right marginal branch (RMB) was present in all specimens, coursing along the right ventricular margin. It originated mainly from the RCA (93.9%), with a minor contribution from the CA in a few *M. tridactyla* (6.1%). Regarding its termination, the RMB reached the apical level in 52.4% of cases and the pre‐apical level in 47.6%, with no post‐apical endings observed. Species‐level variation was evident: *M. tridactyla* showed mostly apical terminations, whereas *T. tetradactyla* and *C. tatouay* were predominantly pre‐apical.

Anastomoses between the interventricular branches (PIVB and SIVB) were evaluated in specimens where both were present (*n* = 77). They occurred in 28.6% of cases, exclusively in Myrmecophagidae, with higher frequencies in *M. tridactyla* (35.4%) and *T. tetradactyla* (38.5%). Additionally, though less common, anastomotic patterns were also observed in both species, involving branches such as RCA–CB in the basal region, or SIVB–LMB, RMB–LMB, PIVB–LMB, and even complex configurations linking PIVB, SIVB, RMB, and LMB in the apical region.

### 3.2 Quantitative descriptive angioarchitecture of the coronary arteries and their branches

The total number of ventricular branches arising from each coronary artery is summarized in Tables 2 and 3. For the PIVB, the number of branches supplying the left ventricle varied significantly among species, ranging from the highest mean in *C. tatouay* (10.00 ± 1.00) to the lowest in *B. crinitus* (2.75 ± 0.96). Branches to the right ventricle were comparatively more homogeneous, though the same species stood out at opposite extremes (*C. tatouay*: 4.67 ± 1.53; *B. crinitus*: 1.25 ± 1.26). Across all species, the left ventricle consistently received a greater number of branches from the PIVB than from the right ventricle. The number of ventricular branches from the CB to the left ventricle was relatively homogeneous across species, with a mean of 4.74 ± 1.74 branches.

**TABLE 2:** Mean and standard deviation of the number of ventricular branches arising from coronary branches, for each ventricle individually, in species of the superorder Xenarthra (*n* = 82).

Species	PIVB (vbLV)	PIVB (vbRV)	CB (vbLV)	SIVB (vbLV)	SIVB (vbRV)
*Myrmecophaga tridactyla* (*n* = 48)	5.49 ± 1.67^b^	2.64 ± 1.17^ab^	5.35 ± 1.66^a^	2.54 ± 1.17^a^	4.92 ± 1.30^a^
*Tamandua tetradactyla* (*n* = 13)	5.08 ± 1.66^bc^	3.08 ± 1.12^ab^	4.77 ± 2.28^a^	1.62 ± 0.87^ab^	3.69 ± 1.25^a^
*Bradypus variegatus* (*n* = 2)	4.50 ± 0.71^bc^	2.50 ± 0.71^ab^	3.50 ± 0.71^a^	1.50 ± 0.71^ab^	2.50 ± 0.71^a^
*Bradypus crinitus* (*n* = 4)	2.75 ± 0.96^c^	1.25 ± 1.26^b^	3.25 ± 1.89^a^	0.75 ± 0.50^b^	3.25 ± 0.96^a^
*Cabassous uncintus* (*n* = 2)	5.50 ± 3.54^ab^	2.50 ± 0.71^ab^	4.00 ± 1.41^a^	1.00 ± 0.00^ab^	2.50 ± 0.71^a^
*Cabassous tatouay* (*n* = 3)	10.00 ± 1.00^a^	4.67 ± 1.53^a^	2.33 ± 0.58^a^	N/A	N/A
*Dasypus novemcinctus* (*n* = 4)	5.50 ± 0.58^b^	2.50 ± 0.58^ab^	6.00 ± 1.15^a^	0.50 ± 0.58^b^	3.50 ± 1.29^a^
*Euphractus sexcinctus* (*n* = 3)	4.00 ± 1.00^bc^	2.00 ± 1.00^ab^	3.00 ± 1.00^a^	N/A	N/A
*Tolypeutes tricinctus* (*n* = 3)	4.00 ± 1.00^bc^	2.00 ± 1.00^ab^	3.67 ± 0.58^a^	1.33 ± 0.58^ab^	2.67 ± 0.58^a^

*Note*: N/A indicates the absence of branches in the specified species. Different letters within the same column indicate significant differences (*p* < 0.05) in the one‐way analysis of variance (ANOVA), complemented by the Tukey post hoc test.

Abbreviations: CB, circumflex branch; LCB, left coronary branch; PIVB, paraconal interventricular branch; SIVB, subsinuosal interventricular branch; vbLV, ventricular branches for Left Ventricle; vbRV, ventricular branches for Right Ventricle.

**TABLE 3:** Mean and standard deviation of the number of branches (atrial and ventricular) originating from the coronary artery and coronary branches, for each ventricle individually, in species of the superorder Xenarthra (*n* = 82).

Species	RCA (abRA)	CB (abLA)	RCA (vbRV)	RCA (vbLV)	CA (vbRV)
*Myrmecophaga tridactyla* (*n* = 48)	4.04 ± 1.01^a^	3.83 ± 1.26^a^	4.54 ± 1.49^a^	0.63 ± 0.94^b^	3.06 ± 1.19^a^
*Tamandua tetradactyla* (*n* = 13)	3.92 ± 0.76^ab^	3.38 ± 1.19^a^	4.92 ± 1.32^a^	1.69 ± 1.49^a^	2.08 ± 0.95^a^
*Bradypus variegatus* (*n* = 2)	3.50 ± 0.71^ab^	3.50 ± 0.71^a^	3.00 ± 1.41^a^	N/A	2.50 ± 0.71^a^
*Bradypus crinitus* (*n* = 4)	2.25 ± 0.50^b^	2.50 ± 0.58^a^	2.75 ± 0.50^a^	0.75 ± 0.50^ab^	2.00 ± 0.82^a^
*Cabassous uncintus* (*n* = 2)	3.50 ± 0.71^ab^	2.50 ± 0.71^a^	2.50 ± 0.71^a^	N/A	1.50 ± 0.71^a^
*Cabassous tatouay* (*n* = 3)	2.67 ± 0.58^ab^	2.33 ± 0.58^a^	4.00 ± 1.00^a^	0.33 ± 0.58^ab^	1.33 ± 0.60^a^
*Dasypus novemcinctus* (*n* = 4)	3.25 ± 0.96^ab^	3.00 ± 0.00^a^	4.00 ± 1.41^a^	N/A	2.00 ± 0.82^a^
*Euphractus sexcinctus* (*n* = 3)	2.33 ± 0.58^ab^	1.67 ± 0.58^a^	4.33 ± 0.58^a^	N/A	1.67 ± 1.15^a^
*Tolypeutes tricinctus* (*n* = 3)	2.67 ± 0.58^ab^	2.33 ± 0.58^a^	3.33 ± 0.58^a^	N/A	1.33 ± 0.58^a^

*Note*: N/A indicates the absence of branches in the respective species. Different letters in the same column indicate a significant difference (*p* < 0.05) in the one‐way analysis of variance (ANOVA) followed by Tukey's post hoc test.

Abbreviations: abLA, atrial branches for Left Atrium; abRA, atrial branches for Right Atrium; CA, conal artery; CB, circumflex branch of the left coronary artery; RCA, right coronary artery; vbLV, ventricular branches for Left Ventricle; vbRV, ventricular branches for Right Ventricle.

Similar to the PIVB, the SIVB also supplied both ventricles, but it contributed more branches to the right ventricle. No significant interspecific differences were observed, although *M. tridactyla* showed the highest mean number of right ventricular branches (4.92 ± 1.30).

The number of ventricular branches originating from the RCA did not differ significantly among species. *T. tetradactyla* showed the highest mean (4.92 ± 1.32). Branches to the left ventricle were absent in some species, and when present, they were consistently fewer than those directed to the right ventricle.

Although smaller than the main coronary branches, the conal artery (CA) consistently gave rise to right ventricular branches in all species examined, with no significant interspecific differences. *M. tridactyla* exhibited the highest mean number (3.06 ± 1.19).

Atrial branches were observed from both the RCA and the CB. The mean number of branches from the RCA ranged from 2.33 ± 0.58 in *E. sexcinctus* to 4.04 ± 1.01 in *M. tridactyla*, while those from the CB ranged from 1.67 ± 0.58 in *E. sexcinctus* to 3.83 ± 1.26 in *M. tridactyla*.

Regarding ventricular branches from the PIVB, most species exhibited a homogeneous distribution, with means ranging from 6.00 ± 1.00 to 8.15 ± 2.41. The only exception was *C. tatouay*, which exhibited a markedly higher mean (14.70 ± 2.31), significantly differing from the other species (Table 4).

**TABLE 4:** Mean and standard deviation of the total number of ventricular branches (bV) originating from the paraconal interventricular branch (PIVB) and the subsinuous interventricular branch (SIVB) in species of the superorder Xenarthra (*n* = 82).

Species	PIVB (bV)	SIVB (bV)	*p*‐value
*Myrmecophaga tridactyla* (*n* = 48)	7.96 ± 2.86^b^	7.46 ± 2.26^a^	0.344
*Tamandua tetradactyla* (*n* = 13)	8.15 ± 2.41^b^	5.31 ± 1.97^b^	0.003*
*Bradypus variegatus* (*n* = 2)	7.00 ± 0.00^b^	4.00 ± 1.41^bc^	0.096
*Bradypus crinitus* (*n* = 4)	4.00 ± 2.16^b^	4.00 ± 1.15^bc^	0.999
*Cabassous uncintus* (*n* = 2)	8.00 ± 4.24^b^	3.50 ± 0.71^bc^	0.277
*Cabassous tatouay* (*n* = 3)	14.70 ± 2.31^a^	0.67 ± 1.15^c^	0.001*
*Dasypus novemcinctus* (*n* = 4)	8.00 ± 0.00^b^	4.00 ± 1.15^bc^	<0.001*
*Euphractus sexcinctus* (*n* = 3)	6.00 ± 1.00^b^	0.67 ± 1.15^c^	0.004*
*Tolypeutes tricinctus* (*n* = 3)	6.00 ± 1.73^b^	4.00 ± 1.00^bc^	0.159

*Note*: Different letters in the same column indicate a significant difference (*p* < 0.05) according to one‐way analysis of variance (ANOVA) followed by Tukey's post hoc test. The * next to the *p*‐value indicates a significant difference in the comparison between the mean number of branches originating from the PIVB and SIVB per species according to Student's *t* test. Both tests considered differences significant when *p* < 0.05.

Abbreviation: rrV, total ventricular branches.

The mean number of ventricular branches from the SIVB varied among species, with *M. tridactyla* showing the highest value (7.46 ± 2.26), while *B. variegatus*, *B. crinitus*, *C. uncintus*, *D. novemcinctus*, and *T. tricinctus* presented lower means (3.50–4.00).

When comparing the total number of branches between the PIVB and the SIVB, significant interspecific differences were found. Four species showed statistical divergence between these vessels: *T. tetradactyla* (*p* = 0.003), *C. tatouay* (*p* = 0.001), *D. novemcinctus* (*p* < 0.001), and *E. sexcinctus* (*p* = 0.004) (Table 4).

Given the larger sample size for *M. tridactyla*, it was possible to analyze this species separately. Using the Student's *t* test, the mean number of ventricular and atrial branches was compared between sexes (males vs. females) and age groups (juveniles vs. adults). No significant differences were detected in either comparison (*p* > 0.05) (Table 5).

**TABLE 5:** Mean and standard deviation of the number of ventricular branches originating from coronary arteries in *Myrmecophaga tridactyla* (*n* = 48), separated by sex and age.

Coronary artery	Adult male (*n* = 17)	Adult female (*n* = 17)	Juvenile male (*n* = 6)	Juvenile female (*n* = 3)	Adult (*n* = 34)	Juvenile (*n* = 9)
PIVB (bV)	7.23 ± 3.06	7.71 ± 2.64	8.17 ± 2.78	7.67 ± 1.53	7.50 ± 2.82	8.00 ± 2.35
PIVB (vbLV)	5.50 ± 1.75	5.24 ± 1.71	5.33 ± 1.75	5.00 ± 1.00	5.36 ± 1.71	5.22 ± 1.48
PIVB (vbRV)	2.25 ± 0.93	2.47 ± 1.12	2.83 ± 1.17	2.67 ± 0.58	2.36 ± 1.03	2.78 ± 0.97
CB (abLA)	3.82 ± 1.13	3.76 ± 1.64	4.00 ± 1.10	4.00 ± 1.00	3.79 ± 1.39	4.00 ± 1.00
CB (vbLV)	4.82 ± 1.13	5.71 ± 1.96	4.33 ± 1.03	4.67 ± 1.15	5.26 ± 1.64	4.44 ± 1.01
SIVB (bV)	8.00 ± 2.55	7.71 ± 2.17	6.67 ± 2.16	5.67 ± 0.58	7.85 ± 2.34	6.33 ± 1.80
SIVB (vbLV)	2.76 ± 1.15	2.71 ± 1.31	2.33 ± 1.21	1.67 ± 0.58	2.74 ± 1.21	2.11 ± 1.05
SIVB (vbRV)	5.24 ± 1.60	5.00 ± 1.06	4.33 ± 1.03	4.00 ± 1.00	5.12 ± 1.34	4.22 ± 0.97
RCA (abRA)	4.00 ± 0.80	4.12 ± 1.17	3.50 ± 1.22	4.33 ± 1.15	4.06 ± 0.98	3.78 ± 1.20
RCA (vbRV)	5.00 ± 1.62	4.59 ± 1.58	4.00 ± 1.26	3.67 ± 0.58	4.79 ± 1.59	3.89 ± 1.05
RCA (vbLV)	0.94 ± 0.97	0.41 ± 0.87	0.50 ± 0.84	0.34 ± 0.58	0.68 ± 0.95	0.45 ± 0.73
CA (vbRV)	3.53 ± 1.23	2.82 ± 1.07	2.83 ± 1.01	3.67 ± 1.53	3.18 ± 1.19	3.11 ± 1.05

Abbreviations: abLA, atrial branches for Left Atrium; abRA, atrial branches for Right Atrium; bV, total ventricular branches; CA, conal artery; CB, circumflex branch of the left coronary artery; PIVB, paraconal interventricular branch; RCA, right coronary artery; SIVB, subsinuosal interventricular branch; vbLV, ventricular branches for Left Ventricle; vbRV, ventricular branches for Right Ventricle.

### 3.3 Coronary ostia

In all examined hearts (*n* = 82), a single ostium for the RCA was identified in the right aortic sinus. Additional openings were also observed in this sinus: the conal artery (CA) ostium in 18 specimens (21.9%), predominantly in *M. tridactyla* (*n* = 17; 35.4%) and in one *T. tetradactyla* (7.7%), and the accessory coronary artery (ACA) ostium in nine specimens (11%), including seven *M. tridactyla* (14.6%) and two *T. tetradactyla* (15.4%).

In the left aortic sinus, a single ostium for the LCA was identified in 72 specimens (87.8%). A double ostium for the LCA was observed in only one *M. tridactyla* (1.2%). The absence of an LCA ostium occurred in four *M. tridactyla* (8.3%), three *T. tetradactyla* (23.1%), one *B. variegatus* (50%), and one *C. uncintus* (50%).

Additional variations were also recorded. A single ostium for the PIVB was identified in seven specimens (8.5%: three *M. tridactyla*, two *T. tetradactyla*, one *B. variegatus*, and one *C. uncintus*), while two distinct ostia for the PIVB were observed in one *T. tetradactyla* (1.2%). For the CB, a single ostium was found in eight specimens (9.7%: four *M. tridactyla*, two *T. tetradactyla*, one *B. variegatus*, and one *C. uncintus*).

When considering both aortic bulb sinuses, most specimens (*n* = 53; 64.6%) exhibited two ostia, including 56.2% of *M. tridactyla*, 53.8% of *T. tetradactyla*, 50% of *B. variegatus* and *C. uncintus*, and 100% of *B. crinitus*, *C. tatouay*, *D. novemcinctus*, *E. sexcinctus*, and *T. tricinctus*. Three ostia were observed in 24 specimens (29.3%), comprising 33.3% of *M. tridactyla*, 46.1% of *T. tetradactyla*, and 50% of *B. variegatus* and *C. uncintus*. Less frequent configurations included four ostia in three specimens (3.6%; 6.2% of *M. tridactyla*) and five ostia in two specimens (2.4%; 4.2% of *M. tridactyla*).

### 3.4 Morphological aspects of coronary distribution

Most specimens (*n* = 61; 74.4%) showed an epicardial course of coronary vessels, while intramyocardial trajectories were observed in 14 hearts (17.1%), and mixed patterns associated with myocardial bridges in seven (8.5%). In one *E. sexcinctus* (33.3%), both intramyocardial and mixed courses co‐occurred. Among the branches with intramyocardial course (*n* = 14), the most frequently involved were the PIVB (92.8%) and SIVB (85.7%), followed by the RCA and LMB (42.8% each), RMB (21.4%), and CB (7.1%). This arrangement was present in 100% of *C. uncintus*, *C. tatouay*, *E. sexcinctus*, and *T. tricinctus*; in 50% of *D. novemcinctus*; and in 7.7% of *T. tetradactyla*. Myocardial bridges (*n* = 7), all located over the PIVB, were identified in *M. tridactyla* (6.5%), *T. tetradactyla* (15.4%), *D. novemcinctus* (25%), and *E. sexcinctus* (33.3%).

### 3.5 Anatomical variations

A total of 11 xenarthran hearts exhibited variations in the origin and/or course of coronary vessels compared to the typical pattern. The SIVB originated from the CB in four cases (5.1%), including *M. tridactyla* (2.1%), *B. crinitus* (25%), and *D. novemcinctus* (50%). In one *C. uncintus* specimen (50%), the PIVB arose directly from the right aortic sinus, adjacent to the RCA, and crossed between the aorta and pulmonary trunk before running along the paraconal interventricular sulcus to the apex. In another variation observed in *M. tridactyla* (2.1%), the CA originated from the right sinus, coursed around the pulmonary trunk, and extended over the paraconal interventricular sulcus to the apex, with complete absence of the PIVB.

Additional compensatory patterns were also identified. In two *C. tatouay* (66.7%) and two *E. sexcinctus* (66.7%), the absence of the SIVB led the LMB to project toward the subsinuosal sulcus, following an intramural course to the pre‐apical region. In one *T. tetradactyla* (7.7%), the absence of the CB was compensated by duplication of the PIVB to supply the left ventricle; in this specimen, the RCA also gave rise to the LMB, which contributed to left ventricular irrigation.

### 3.6 Coronary dominance

#### 3.6.1 Criterion I: Origin of the interventricular branches, directly or indirectly, from the aortic bulb sinus (left, right, or both)

Most of the hearts analyzed (*n* = 72; 92.3%) exhibited a balanced pattern, characterized by the SIVB arising from the right aortic sinus and the PIVB from the left. This arrangement was consistent across all specimens of *T. tetradactyla*, *B. variegatus*, and *T. tricinctus*, as well as in 95.8% of *M. tridactyla*, 75% of *B. crinitus*, 50% of *C. uncintus* and *D. novemcinctus*, and 33.3% of *C. tatouay* and *E. sexcinctus*.

A left coronary pattern was identified in four hearts (5.1%), including one *M. tridactyla* (2.1%), one *B. crinitus* (25%), and two *D. novemcinctus* (50%). In these cases, both interventricular branches (PIVB and SIVB) originated from the left coronary sinus.

A right coronary pattern occurred in only two hearts (2.6%). One *M. tridactyla* specimen presented the PIVB arising directly from the right aortic sinus, while in one *C. uncintus*, the CA, originating from the right sinus, replaced the absent PIVB by coursing toward the left surface and becoming an interventricular branch over the paraconal sulcus.

Finally, four hearts (4.9%) could not be classified under this criterion due to the absence of the SIVB, specifically in two *C. tatouay* and two *E. sexcinctus*.

#### 3.6.2 Criterion II: Total number of ventricular branches originating directly from each aortic bulb sinus (left or right), with predominantly left, right, or balanced blood flow

**FIGURE 4:**
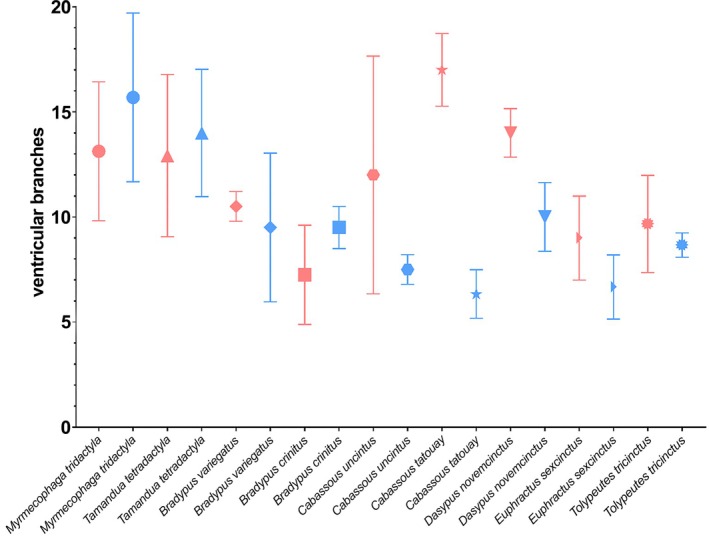
Graph showing the mean and standard deviation of the total number of ventricular branches originating from the left and right sides of the aortic bulb, by species of the Xenarthra superorder (*n* = 82). The blue color represents the blood flow originating from the right aortic sinus, and the red color represents the blood flow originating from the left aortic sinus. Each geometric symbol represents a species.

Analysis of ventricular branches originating from the aortic bulb sinuses (Table 6 and Figure 4) showed that most xenarthran species (six of nine) exhibited a balanced pattern, characterized by the absence of significant differences between left‐ and right‐sided origins. This configuration was observed in *T. tetradactyla*, *B. variegatus*, *B. crinitus*, *C. uncintus*, *E. sexcinctus*, and *T. tricinctus*.

**TABLE 6:** Mean and standard deviation of the total number of ventricular branches from the left and right sides of the aortic bulb, by species of the superorder Xenarthra (*n* = 82).

Species	Left flow	Right flow	*p*‐value
	PIVB (bV) + CB (bV)	RCA (bV) + SIVB (bV) + CA (bV)	
*Myrmecophaga tridactyla* (*n* = 48)	13.10 ± 3.31^ab^	15.47 ± 4.02^a^	0.001*
*Tamandua tetradactyla* (*n* = 13)	12.90 ± 3.86^abc^	14.00 ± 3.03^ab^	0.437
*Bradypus variegatus* (*n* = 2)	10.50 ± 0.71^abc^	9.50 ± 3.54^bc^	0.733
*Bradypus crinitus* (*n* = 4)	7.25 ± 2.36^c^	9.50 ± 1.00^bc^	0.130
*Cabassous uncintus* (*n* = 2)	12.00 ± 5.66^abc^	7.50 ± 0.71^bc^	0.380
*Cabassous tatouay* (*n* = 3)	17.00 ± 1.73^a^	6.33 ± 1.15^c^	<0.001*
*Dasypus novemcinctus* (*n* = 4)	14.00 ± 1.15^abc^	10.00 ± 1.63^abc^	0.007*
*Euphractus sexcinctus* (*n* = 3)	9.00 ± 2.00^abc^	6.67 ± 1.53^c^	0.184
*Tolypeutes tricinctus* (*n* = 3)	9.67 ± 2.31^abc^	8.67 ± 0.58^bc^	0.507

*Note*: The * next to the *p*‐value indicates a significant difference (*p*‐value <0.05 in the unpaired Student's *t* test). Different letters in the same column indicate significant differences in one‐way analysis of variance (ANOVA), complemented by the Tukey post hoc test.

Abbreviations: bV, total ventricular branches; CA, conal artery; CB, circumflex branch of the left coronary artery; PIVB, paraconal interventricular branch; RCA, right coronary artery; SIVB, subsinuosal interventricular branch.

Two species presented a left coronary pattern: *C. tatouay* (*p* < 0.001) and *D. novemcinctus* (*p* = 0.007). In contrast, *M. tridactyla* showed a right coronary pattern (*p* = 0.001). Notably, the highest totals of ventricular branches were recorded in *C. tatouay* (mean 17.00 ± 1.73 from the left sinus) and *M. tridactyla* (mean 15.47 ± 4.02 from the right sinus).

#### 3.6.3 Criterion III: Total number of ventricular branches directed to each ventricle (right or left)

**FIGURE 5:**
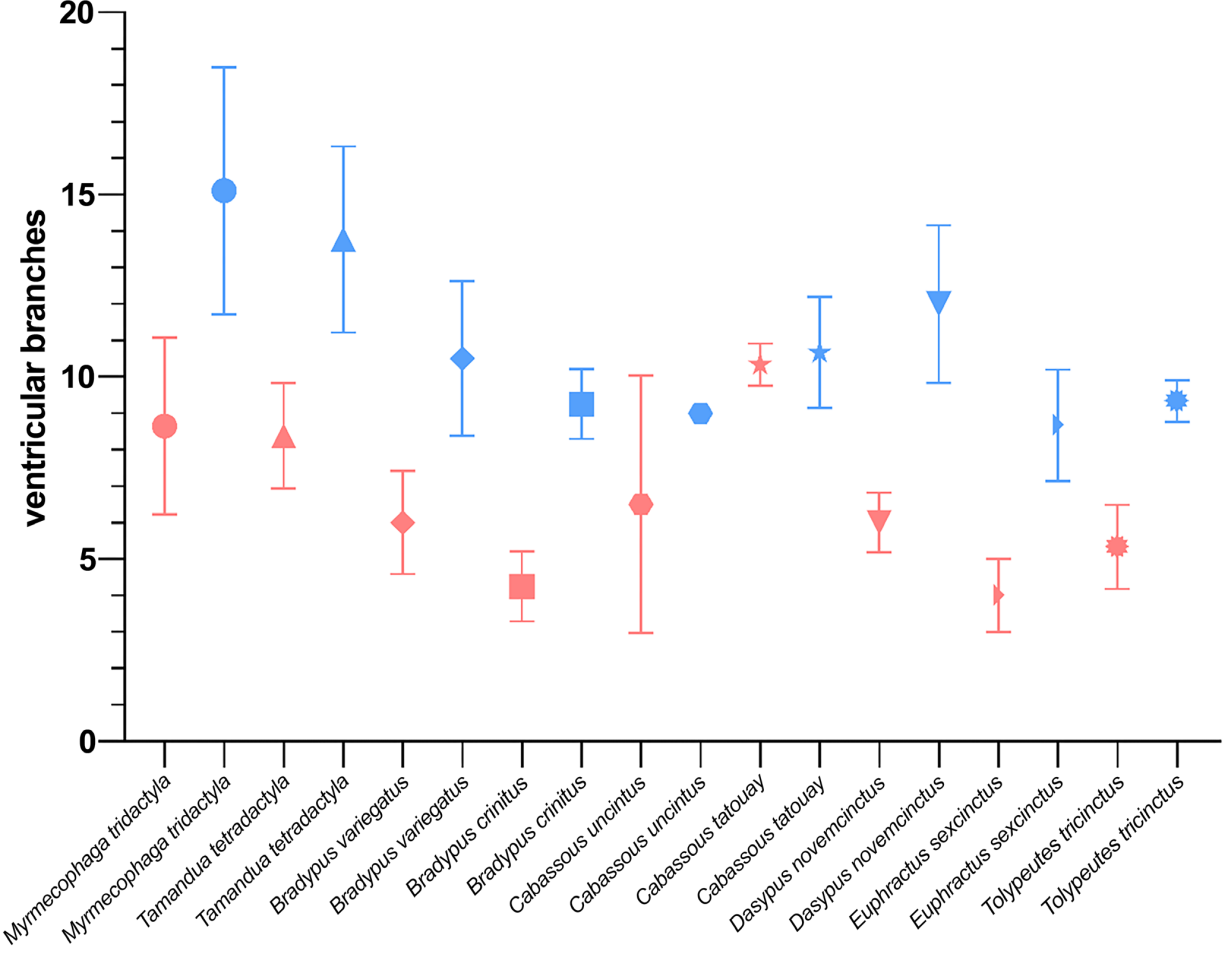
Graph showing the mean and standard deviation of the total number of ventricular branches supplying the left ventricle (LV) and right ventricle (RV), separated by species of the superorder Xenarthra (*n* = 82). The blue color represents the ventricular branches supplying the right ventricle, and the red color represents the ventricular branches supplying the left ventricle. Each geometric symbol represents a different species.

When ventricular branches were grouped by destination (LV vs. RV), most species (six of nine) exhibited a right coronary pattern (Table 7 and Figure 5). This arrangement was consistent across both anteaters (*M. tridactyla*, 15.10 ± 3.39, *p* < 0.001; *T. tetradactyla*, 13.80 ± 2.55, p < 0.001), three armadillos (*D. novemcinctus*, 12.00 ± 2.16, *p* = 0.002; *E. sexcinctus*, 8.67 ± 1.53, *p* = 0.011; *T. tricinctus*, 9.33 ± 0.58, *p* = 0.006), and one sloth (*B. crinitus*, 9.25 ± 0.96, *p* < 0.001).

**TABLE 7:** Mean and standard deviation of the number of branches destined to the left and right ventricles in Xenarthras.

Species	Ventricular branches to the left ventricle	Ventricular branches to the right ventricle	*p*‐value
*Myrmecophaga tridactyla* (*n* = 48)	8.65 ± 2.43^ab^	15.10 ± 3.39^a^	<0.001*
*Tamandua tetradactyla* (*n* = 13)	8.38 ± 1.45^ab^	13.80 ± 2.55^ab^	<0.001*
*Bradypus variegatus* (*n* = 2)	6.00 ± 1.00^ab^	10.50 ± 2.12^ab^	0.130
*Bradypus crinitus* (*n* = 4)	4.25 ± 0.96^bc^	9.25 ± 0.96^b^	<0.001*
*Cabassous uncintus* (*n* = 2)	6.50 ± 3.54^ab^	9.00 ± 0.00^b^	0.427
*Cabassous tatouay* (*n* = 3)	10.30 ± 0.58^a^	10.57 ± 1.53^ab^	0.742
*Dasypus novemcinctus* (*n* = 4)	6.00 ± 0.82^ab^	12.00 ± 2.16^ab^	0.002*
*Euphractus sexcinctus* (*n* = 3)	4.00 ± 1.00^bc^	8.67 ± 1.53^b^	0.011*
*Tolypeutes tricinctus* (*n* = 3)	5.33 ± 1.15^bc^	9.33 ± 0.58^b^	0.006*

*Note*: The * next to the *p*‐value indicates a significant difference (*p* < 0.05 in the unpaired Student's *t* test). Different letters in the same column indicate a significant difference in the one‐way analysis of variance (ANOVA), complemented by the Tukey post hoc test.

Within this framework, *M. tridactyla* had the highest mean number of branches directed to the RV (15.10 ± 3.39), while *E. sexcinctus* exhibited the lowest mean number to the LV (4.00 ± 1.00).

## 4 DISCUSSION

### 4.1 Potential factors underlying coronary diversity in Xenarthra

The aim of this Discussion is to interpret the coronary patterns observed in the xenarthran hearts of this study, emphasizing the intragroup diversity revealed by our data and comparing these findings with previous reports in other mammalian species.

The comparative analysis of xenarthran specimens revealed that some morphological traits tend to cluster by family, suggesting a possible phylogenetic component, and providing insights into both conserved and divergent traits within the superorder (Delsuc et al., 2002). A key finding is the remarkable prevalence of right coronary artery (RCA) dominance, responsible for supplying the subsinuous interventricular branch in 94.9% of the specimens. This consistent pattern across all studied families suggests this feature is ancestral for the superorder, having been maintained throughout its diversification. Similarly, the distribution of vessels was predominantly epicardial (74.4%). This prevalence suggests that xenarthrans retain a configuration consistent with that described for monotremes (Dowd, 1969) rather than the intramyocardial variant observed in marsupials (Dowd, 1974).

While these unifying traits are evident, our findings also reveal distinct patterns that align with the major phylogenetic clades. For instance, the Cingulata clade (armadillos) shows a trend toward a higher number of branches for the left ventricle, with species like *Cabassous tatouay* and *Dasypus novemcinctus* presenting a greater average number of branches. In contrast, the sister clade, Pilosa (anteaters and sloths), displays a more constrained branching pattern, as observed in *Bradypus* and *Tamandua*.

Furthermore, some specific morphological traits appear to be phylogenetically restricted. For example, anastomoses between the paraconal and subsinuous interventricular branches were identified exclusively in Myrmecophagidae. This anatomical differentiation in vascular complexity between the clades mirrors their long evolutionary divergence and indicates that while the fundamental coronary design is conserved, the fine‐scale morphology has evolved along separate paths.

This tendency of species from the same family to share coronary features is consistent with previous observations in other mammalian groups, such as carnivores and cervids, where family‐level clades exhibit characteristic configurations of coronary dominance (Viotto‐Souza et al., 2023; Zaniboni et al., 2021). These results support the interpretation that at least part of the diversity observed among xenarthran specimens of this study may reflect evolutionary history and lineage‐specific constraints.

In addition to phylogenetic signal, our data may suggest that body size and ventricular workload can also influence coronary architecture. *M. tridactyla*, the largest species examined, exhibited the highest mean number of ventricular branches originating from the subsinuous interventricular branch and was the only xenarthran in which two distinct SIVBs were observed. This finding aligns with quantitative studies in mammals demonstrating coronary arterial dimensions and branching scale with myocardial mass and perfusion demand (Choy & Kassab, 2008). In contrast, smaller xenarthran species, such as *C. tatouay* and *E. sexcinctus*, occasionally lacked the SIVB entirely, a condition rarely observed in large‐bodied mammals.

Another aspect that may explain the observed heterogeneity is metabolic rate and ecological habit. Sloths of the genus *Bradypus* (Bradypodidae) exhibited lower mean numbers of paraconal and subsinuous branches compared to anteaters and armadillos, consistent with their markedly reduced metabolic rate and heart rate, which are among the lowest recorded for mammals of comparable body mass (Cliffe et al., 2018). This simplified coronary branching pattern may represent an adaptation to the species' low energetic demands and sedentary lifestyle. In contrast, anteaters such as *M. tridactyla* and *T. tetradactyla*, which engage in long foraging bouts and exhibit high‐intensity bursts of digging activity, display more complex and redundant coronary arrangements, including the presence of intramyocardial courses and additional ventricular branches.

Armadillos, many of which are fossorial, also demonstrated variability in coronary distribution that could be linked to the intermittent increases in intrathoracic pressure associated with digging. Together, these findings suggest that functional and ecological pressures interact with evolutionary history to shape the diversity of coronary arteries in Xenarthra. Many armadillo species (family Dasypodidae) are fossorial, engaging in regular digging behavior for foraging and shelter (Clerici et al., 2018; Marshall et al., 2021). Such activity involves powerful forelimb flexion and extension, along with repeated compression of the thoracic cavity, particularly when driving soil with forceful limb strokes (Vizcaíno & Milne, 2002). These intermittent increases in intrathoracic pressure can transiently compromise venous return and cardiac filling, a phenomenon analogous to the human Valsalva maneuver. To ensure consistent myocardial perfusion during these maneuvers, a more variable or redundant coronary architecture may confer functional resilience, for example, through alternative branching routes or intramyocardial protection.

In our sample, armadillos, such as *C. tatouay* and *E. sexcinctus*, displayed notable variability in coronary distribution, including the occasional absence of the subsinuous interventricular branch (SIVB) or atypical origins of ventricular branches. This anatomic variability may represent an adaptive response to the physical demands of fossorial locomotion and thoracic loading. In other words, the coronary network in these species may have evolved greater flexibility or redundancy to maintain perfusion stability under episodic high intrathoracic pressures associated with digging.

These findings offer important insights into the coronary anatomy of Xenarthra and underscore the need for more comprehensive studies to gain a thorough understanding of cardiovascular adaptations within this superorder. Expanding the number of specimens and species, particularly from the families Bradypodidae and Dasypodidae, will be essential to better characterize coronary distribution and variation in circulation types. The focus of the present study was to document intragroup variation in coronary artery morphology, rather than to establish the distinctiveness of Xenarthra within a broader evolutionary framework. Although considerable diversity was identified among the species examined, a formal phylogenetic analysis including sister groups (e.g., Afrotheria) and outgroups (other placental or non‐placental mammals) would be necessary to determine whether the observed patterns represent synapomorphies of Xenarthra or lineage‐specific features. While such an analysis was beyond the scope of this work, our results provide a valuable baseline that can be integrated into future comparative and phylogenetically informed studies.

### 4.2 Comparative overview of coronary patterns across mammals

#### 4.2.1 Main coronary trunks (LCA and RCA)

In our xenarthran sample, a common trunk of the left coronary artery (LCA) was present in 73 hearts (89%). Similar near‐universal occurrence has been described in domestic mammals, including dogs, cats, cattle, and pigs (Correia‐Oliveira, Moraes, et al., 2014; Monfared et al., 2013; Moura Junior et al., 2008; Oliveira et al., 2010), and in some wild species such as *Sus scrofa*, *Puma concolor*, *Mazama gouazoubira*, *Mus musculus*, *Cuniculus paca*, and *Oryctolagus cuniculus* (Ávila et al., 2009; Correia‐Oliveira, Oliveira, et al., 2014; Ribeiro et al., 2014; Viotto‐Souza et al., 2017; Yoldas et al., 2010; Zaniboni et al., 2021). Lower frequencies have been reported for *Myrmecophaga tridactyla* (83.3–95%) (Cruvinel et al., 2019; Santos et al., 2020) and *Sapajus apella* (84%) (Srour, 2011). When absent, the left sinus still originated the paraconal interventricular (PIVB) and circumflex (CB) branches (Ávila et al., 2009; Correia‐Oliveira, Moraes, et al., 2014; Correia‐Oliveira, Oliveira, et al., 2014; Cruvinel et al., 2019; Monfared et al., 2013; Moura Junior et al., 2008; Oliveira et al., 2010; Ribeiro et al., 2014; Santos et al., 2020; Viotto‐Souza et al., 2017; Yoldas et al., 2010; Zaniboni et al., 2021).

The right coronary artery (RCA) was consistently observed in all xenarthran specimens, as in most domestic and wild mammals (Ávila et al., 2009; Correia‐Oliveira, Moraes, et al., 2014; Correia‐Oliveira, Oliveira, et al., 2014; Monfared et al., 2013; Moura Junior et al., 2008; Oliveira et al., 2010; Ribeiro et al., 2014; Santos et al., 2020; Viotto‐Souza et al., 2017; Yoldas et al., 2010; Zaniboni et al., 2021). An exception was reported by Cruvinel et al. (2019), who found the RCA in only half of their *M. tridactyla* sample, with the right sinus directly giving rise to the subsinuous interventricular (SIVB) and right marginal branches (RMB).

#### 4.2.2 Interventricular and circumflex branches

The PIVB and CB were present in 98.8% of the analyzed xenarthran specimens, absent only in two Myrmecophagidae. In such cases, compensatory arrangements were observed, such as replacement of the PIVB by a conal artery in *M. tridactyla* and duplication of the PIVB in *T. tetradactyla*. Similar consistency of both branches has been reported in domestic mammals (*C. lupus familiaris*, *F. catus*, *B. taurus*, *S. scrofa domesticus*) and in wild taxa such as *S. scrofa*, *C. paca*, *M. musculus*, *M. gouazoubira*, *P. concolor*, and *S. apella* (Ávila et al., 2009; Correia‐Oliveira, Moraes, et al., 2014; Monfared et al., 2013; Moura Junior et al., 2008; Oliveira et al., 2010; Ribeiro et al., 2014; Srour, 2011; Viotto‐Souza et al., 2017; Yoldas et al., 2010; Zaniboni et al., 2021).

The SIVB was identified in 78 xenarthrans, predominantly originating from the RCA (94.9%), corroborating findings in *C. paca* (Ávila et al., 2009), *P. concolor* (Viotto‐Souza et al., 2017), *Camelus dromedarius* (Yuan et al., 2009), *B. variegatus* (Albuquerque et al., 2022), and *T. tetradactyla* (Pinheiro et al., 2014). Lower frequencies of RCA origin have been reported in *M. tridactyla* (41.7%, Cruvinel et al., 2019; 5%, Santos et al., 2020), *F. catus* (33.3%, Biasi et al., 2012), and *C. lupus familiaris* (6.4%, Andretto et al., 1973). In four xenarthrans (5.1%), the SIVB arose from the CB, as consistently seen in dogs and cattle (Biasi et al., 2013; Correia‐Oliveira, Moraes, et al., 2014; Moore, 1930; Oliveira et al., 2011), in *M. gouazoubira* (Zaniboni et al., 2021), and occasionally in *M. tridactyla* (Cruvinel et al., 2019). Absence of the SIVB, found in four xenarthrans (4.9%; *C. tatouay* and *E. sexcinctus*), parallels the 100% absence reported for *M. musculus* (Yoldas et al., 2010) and *O. cuniculus domesticus* (Bahar et al., 2007).

#### 4.2.3 Septal branch and courses

The septal branch (SB) originated from the PIVB in 75.6% (*n* = 62) of the hearts analyzed, similar to *O. cuniculus domesticus* (75%) (Bahar et al., 2007) but lower than in *P. concolor* (100%) (Viotto‐Souza et al., 2017) and *M. gouazoubira* (87.5%) (Zaniboni et al., 2021). In dogs, the frequency was lower (48–60%) (Donald & Essex, 1954; Noestelthaller et al., 2007). In three *M. tridactyla* (6.2%) and one *B. variegatus* (50%), the SB originated from the CB, a pattern also reported in one of 125 dogs examined by Donald and Essex (1954).

The PIVB, SIVB, and CB followed their homonymous grooves in 97.6% (*n* = 80), 98.7% (*n* = 77), and 100% (*n* = 81) of the specimens sampled in this study, respectively, consistent with previous descriptions in carnivorans, bovids, suids, lagomorphs, and xenarthrans (Ávila et al., 2009; Correia‐Oliveira, Moraes, et al., 2014; Monfared et al., 2013; Moura Junior et al., 2008; Oliveira et al., 2010; Pinheiro et al., 2014; Santos et al., 2020; Viotto‐Souza et al., 2017).

Among specimens with the SIVB, apical termination predominated (60.2%; *n* = 47). Apical was likewise the majority in *T. tetradactyla* (75%; Pinheiro et al., 2014), *C. paca* (100%; Ávila et al., 2009), *P. concolor* (66.6%; Viotto‐Souza et al., 2017), and *S. scrofa domesticus* (43.3%; Moura Junior et al., 2008), whereas pre‐apical termination predominated in *F. catus* (79%; Monfared et al., 2013) and *C. lupus familiaris* (70%; Oliveira et al., 2010). For the PIVB, apical termination was most common in our series (87.6%; *n* = 71) and in *T. tetradactyla* (100%; Pinheiro et al., 2014), *C. paca* (100%; Ávila et al., 2009), and *P. concolor* (83.3%; Viotto‐Souza et al., 2017), but was less frequent in *C. lupus familiaris* (56.7%; Oliveira et al., 2010), *F. catus* (48.7%; Monfared et al., 2013), and *S. scrofa domesticus* (43.3%; Moura Junior et al., 2008).

#### 4.2.4 Marginal branches and accessory arteries

A terminological issue must be noted: the CA and ACA are not included in the *Nomina Anatomica Veterinaria* (ICVGAN, 2017), which often leads to their misinterpretation as simple ventricular branches. In carnivorans, the ACA has occasionally been reported arising near the RCA and terminating in the conus arteriosus (Evans & De Lahunta, 2013; Getty, 1986). In humans, a comparable “third coronary artery” may originate from the aorta or RCA (Schlesinger et al., 1949), supplying the conus arteriosus and right ventricle (Agustín et al., 2010; Olabu et al., 2007; Srour, 2011; Yuan et al., 2009). In our series, the ACA was consistently smaller and restricted to the space between the aortic bulb and pulmonary trunk, while the CA was larger, encircling the conus arteriosus and giving off right ventricular branches.

In Myrmecophagidae, the CA arose from the right sinus in 21.9% of specimens, while in 78.1% it originated from the RCA. Aortic origin was also reported in M. tridactyla (Cruvinel et al., 2019), whereas in *Mus musculus* it was exclusively from the RCA (Yoldas et al., 2010). Comparable variability has been described in dogs (26% direct sinus origin) (Büll & Martins, 2002), in *F. catus* and *O. cuniculus* (predominantly RCA, with occasional sinus origin) (Aksoy & Karadağ, 2002), and in camels and pigs, where dual origins from RCA and PIVB were recorded (Ribeiro et al., 2014; Yuan et al., 2009). The ACA occurred in 14.6% of xenarthrans, similar to frequencies in dogs (20%) (Büll & Martins, 2002; Moore, 1930) and in *P. concolor* (50%) (Viotto‐Souza et al., 2017).

The LMB was present in all xenarthrans, originating mainly from the CB (91.5%), with rare deviations such as one *T. tetradactyla* in which it arose from the RCA. This predominance parallels reports in dogs, pigs, rabbits, and primates (Bahar et al., 2007; Moura Junior et al., 2008; Oliveira et al., 2011; Srour, 2011), though lower in cats (89–97%) (Monfared et al., 2013; Rodrigues, 1997) and in *P. concolor* (83.3%) (Viotto‐Souza et al., 2017). The RMB was likewise universal, arising mostly from the RCA (93.9%), consistent with patterns described in pigs, cats, and *P. concolor* (Monfared et al., 2013; Moura Junior et al., 2008; Viotto‐Souza et al., 2017).

#### 4.2.5 Ostia variations

A single ostium for the LCA in the left aortic sinus was identified in 87.8% of specimens, similar to *M. tridactyla* (83.3%; Cruvinel et al., 2019), dogs (89%; Barszcz et al., 2023), and cats (98%; Barszcz et al., 2017). Double ostia for the LCA were less frequent (1.2%), also reported in dogs (9%; Barszcz et al., 2023), cats (3.2%; Barszcz et al., 2017), and bison (18.5%; Barszcz et al., 2020). Additional left sinus variations included single ostia for the PIVB (8.5%) or for the CB (9.7%), patterns likewise described in *M. tridactyla* (Cruvinel et al., 2019), dogs (Barszcz et al., 2023; Büll & Martins, 2002; Noestelthaller et al., 2007), and cats (Barszcz et al., 2017). An unusual case in *T. tetradactyla* presented two distinct PIVBs, a configuration not previously reported, while Santos et al. (2020) noted one specimen of *M. tridactyla* with separate ostia for the LCA and PIVB.

The conal artery (CA) ostium was observed in 21.9% of Myrmecophagidae, compared to 50% in *Saimiri sciureus* (Colborn, 1966, as cited in Durán et al., 2007) and 8% in pigs (*S. scrofa domesticus*; Berg, 1962, as cited in Durán et al., 2007). The accessory coronary artery (ACA) ostium occurred in 10.9% of xenarthrans, all Myrmecophagidae, with similar prevalences in horses (Pereira, Seyfert, et al., 2022), pigs, and sheep (Pereira, Prates, et al., 2022), but higher in dogs (Barszcz et al., 2023) and especially European bison (Barszcz et al., 2020). In *M. tridactyla*, ACA ostia were also recorded by Santos et al. (2020), though without frequency data.

#### 4.2.6 Intramyocardial courses and myocardial bridges

In our series, 17.1% of specimens exhibited intramyocardial trajectories in different branches (PIVB, SIVB, RCA, LMB, RMB, and CB). Comparable conditions have been documented in other mammals, with all coronary branches intramyocardial in *Mesocricetus auratus* (Sans‐Coma et al., 1993), frequent occurrence in *Mazama gouazoubira*, notably 58.3% in the SIVB (Zaniboni et al., 2021), and rare cases in *Blastocerus dichotomus* (Machado et al., 2002).

Myocardial bridges were detected in 8.5% of the xenarthrans analyzed in this study, always over the PIVB, a markedly lower prevalence than in domestic species such as dogs (62–77%) (Farias et al., 2016; Santos et al., 2022), pigs (98%) (Gaggini et al., 2011), cattle (94–100%) (Severino et al., 1997; Severino & Bombonato, 1992), and sheep (96–100%) (Aksoy et al., 2018; Cruz et al., 2007; Melo et al., 2010). High frequencies were also described in mules (Ribeiro et al., 2009), wild suids (Nocetti et al., 2002), pacas (Ávila et al., 2009), and cervids (Zaniboni et al., 2021). In humans, bridges are likewise common and usually involve the PIVB of the left coronary artery (Loukas et al., 2006).

Although often clinically silent, myocardial bridges in humans may underlie arrhythmias, angina, infarction, or sudden death, while other hypotheses suggest physiological benefits, such as enhancing venous return or offering protection against atherosclerosis (Kosiński & Grzybiak, 2001).

#### 4.2.7 Atypical patterns of coronary origin and course

According to Angelini (2007), coronary “anomalies” may involve origin and course, intrinsic pathways, or termination patterns. In a comparative anatomical framework, however, these are better understood as atypical rather than pathological configurations. In our series, two cases of atypical origin and course were identified. In one *Cabassous unicinctus*, the PIVB originated from the right aortic sinus, adjacent to the RCA, and followed an interarterial path. In another case, a *Myrmecophaga tridactyla* specimen showed the PIVB arising from the right sinus and following a prepulmonary (circumpulmonary) trajectory before reaching the interventricular sulcus. In humans, this pattern has been linked to subvalvular pulmonary stenosis and, more rarely, to tetralogy of Fallot (Buchanan, 1990; Kim et al., 2006; Shriki et al., 2012).

### 4.3 Coronary dominance patterns under different criteria

#### 4.3.1 Criterion I (origin of interventricular branches)

The predominant coronary pattern in our xenarthran series was the balanced type, observed in 92.3% of specimens (*n* = 72). This aligns with reports in wild felids such as *Puma concolor*, *Panthera leo*, and *P. pardus* (Viotto‐Souza et al., 2017; Schiller, 1957; Zhang et al., 2008), as well as in *Cuniculus paca* and *Camelus bactrianus* (Ávila et al., 2009; Yuan et al., 2009). In xenarthrans, similar frequencies have been described in *Myrmecophaga tridactyla* (Cruvinel et al., 2019) and *Tamandua tetradactyla* (Pinheiro et al., 2014), though Santos et al. (2020) reported it in only one of 12 anteaters examined.

A left coronary pattern occurred in 5.1% of specimens (*n* = 4), including one *M. tridactyla*, one *Bradypus crinitus*, and two *Dasypus novemcinctus*. Santos et al. (2020) also recorded left dominance in most *T. tetradactyla*. Among other mammals, this is the prevailing pattern in dogs, cattle, and cervids (Biasi et al., 2013; Correia‐Oliveira, Moraes, et al., 2014; Moore, 1930; Oliveira et al., 2011; Zaniboni et al., 2021).

Right dominance was rare, identified in only 2.6% of our material (*n* = 2). A comparable case was described in *T. tetradactyla* by Pinheiro et al. (2014), although only the PIVB origin was detailed.

Overall, the predominance of the balanced coronary pattern in Xenarthra, as analyzed in this study, suggests a conserved arrangement within the group. However, the occurrence of left and right dominance in specific species indicates that coronary distribution is not strictly uniform but may be shaped by lineage‐specific trajectories, ventricular workload, and/or ecological demands.

#### 4.3.2 Criterion II (distribution of ventricular branches)

Analysis by this method again revealed a predominance of the balanced pattern (*n* = 6 species), reinforcing the results from criterion I. Nonetheless, species‐specific tendencies were evident. The left coronary type in *C. tatouay* and *D. novemcinctus* was associated with the absence of the SIVB and the presence of intramyocardial trajectories. By contrast, *M. tridactyla* exhibited a right coronary pattern, seemingly related to the high number of ventricular branches supplied by the CA. However, no significant differences were detected between the PIVB and SIVB branch numbers in this species.

#### 4.3.3 Criterion III (total ventricular branch counts)

Using this approach, right coronary predominance was identified in six xenarthran species (*M. tridactyla*, *T. tetradactyla*, *D. novemcinctus*, *E. sexcinctus*, *T. tricinctus*, and *C. crinitus*). This interpretation is supported by the higher number of branches arising from the CA and other arteries supplying the right ventricle, compared with those directed to the left ventricle. Conversely, species such as *B. variegatus*, *C. unicinctus*, and *C. tatouay*, which displayed a balanced coronary pattern, had fewer branches originating from the CA.

In conclusion, the right sinus of the aortic bulb plays a key role by originating the right coronary, conal, and accessory coronary arteries, while the left sinus generally gives rise to the common trunk of the left coronary artery, which divides into the paraconal interventricular and circumflex branches. Most species showed epicardial pathways, although intramyocardial courses and myocardial bridges were more frequent in armadillos. Coronary dominance was predominantly balanced, yet criterion III revealed a tendency toward right dominance in several species. These patterns combine conserved ancestral traits, such as the prevalence of epicardial distribution and right coronary supply of the subsinuosal branch, with lineage‐specific and ecological adaptations. Simplified branching in sloths aligns with their low metabolic rate, whereas anteaters and fossorial armadillos exhibited greater variability and redundancy, likely reflecting higher cardiovascular demands. Overall, the coronary anatomy observed in the xenarthrans of our sample reflects both phylogenetic history and ecological pressures, highlighting the evolutionary distinctiveness of this group.

## AUTHOR CONTRIBUTIONS

**Wilson Viotto‐Souza:** Investigation; writing – original draft; writing – review and editing; methodology; validation; visualization. **André Luiz Quagliatto Santos:** Funding acquisition; supervision; project administration; writing – review and editing. **Marcelo Abidu‐Figueiredo:** Writing – review and editing; supervision. **Thaís Aparecida Silva:** Methodology; investigation. **Rodrigo Ananias Barreiros Silva:** Investigation; methodology. **Paulo de Souza‐Junior:** Conceptualization; supervision; project administration; writing – review and editing; formal analysis.

## CONFLICT OF INTEREST STATEMENT

The authors declare no conflicts of interest.

## References

[ar70073-bib-0001] Agustín, J. A. , Marcos‐Alberca, P. , Hernández‐Antolín, R. , Vilacosta, I. , Pérez de Isla, L. , Rodríguez, E. , Macaya, C. , & Zamorano, J. (2010). Circulación colateral de arteria conal a descendente anterior: valoración con tomografía coronaria multidetector. Revista Española de Cardiología, 63, 347–351. 10.1016/S0300-8932(10)70094-4 20196996

[ar70073-bib-0002] Aksoy, G. , & Karadağ, H. (2002). An anatomic investigation on the heart and coronary arteries in the domestic cat and white New Zealand rabbits. Eurasian Journal of Veterinary Sciences, 18(1), 33–40.

[ar70073-bib-0003] Aksoy, G. , Özüdoğru, Z. , & Özdemir, D. (2018). A macroanatomic investigation of the coronary arteries and myocardial bridges in Awassi sheep. Eurasian Journal of Veterinary Sciences, 34(3), 171–177. 10.15312/EurasianJVetSci.2018.192

[ar70073-bib-0004] Albuquerque, P. V. , Mesquita, E. P. , Alcântara, S. F. , Miranda, M. E. L. C. , Andrade, G. P. , Amorim Júnior, A. A. , & Amorim, M. J. A. A. L. (2022). External macroscopic anatomy of the Bradypus variegatus heart. Arquivo Brasileiro de Medicina Veterinária e Zootecnia, 74(5), 814–824. 10.1590/1678-4162-12562

[ar70073-bib-0005] Anacleto, T. C. S. , Miranda, F. , Medri, I. , Cuellar, E. , Abba, A. M. , & Superina, M. (2014). Priodontes maximus. The IUCN red list of threatened species 2014: e.T18144A47442343. 10.2305/IUCN.UK.2014-1.RLTS.T18144A47442343

[ar70073-bib-0006] Andretto, R. , Borelli, V. , & Fernandes Filho, A. (1973). Sobre a origem do ramus descendens subsinuosus em cães. Brazilian Journal of Veterinary Research and Animal Science, 10, 5–10. 10.11606/issn.2318-3659.v10i1p5-10

[ar70073-bib-0007] Angelini, P. (2007). Coronary artery anomalies. Circulation, 115(10), 1296–1305. 10.1161/CIRCULATIONAHA.106.618082 17353457

[ar70073-bib-0008] Ávila, B. H. P. , Machado, M. R. F. , Gerbasi, S. H. B. , & Oliveira, F. S. (2009). As artérias coronárias da paca (Agouti paca Linnaeus 1766). Biotemas, 22, 159–162. 10.5007/2175-7925.2009v22n4p159

[ar70073-bib-0009] Bahar, S. , Ozdemir, V. , Eken, E. , & Tipirdamaz, S. (2007). The distribution of the coronary arteries in the angora rabbit. Anatomia, Histologia, Embryologia, 36, 321–327. 10.1111/j.1439-0264.2007.00770.x 17845219

[ar70073-bib-0010] Banchi, A. (1904). Morfologia delle arteriae coronáriae cordis. Italian Journal of Anatomy and Embryology, 3, 87–164.

[ar70073-bib-0011] Barszcz, K. , Goździewska‐Harłajczuk, K. , Czopowicz, M. , Chłopecka, M. , Polguj, M. , & Klećkowska‐Nawrot, J. (2023). Morphometry and topography of the coronary ostia in the dog. Journal of Veterinary Research, 67(3), 471–478. 10.2478/jvetres-2023-0054 37786844 PMC10541670

[ar70073-bib-0012] Barszcz, K. , Kupczyńska, M. , Polguj, M. , Klećkowska‐Nawrot, J. , Janeczek, M. , Goździewska‐Harłajczuk, K. , Dzierzęcka, M. , & Janczyk, P. (2017). Morphometry of the coronary ostia and the structure of coronary arteries in the shorthair domestic cat. PLoS One, 12, e0186177. 10.1371/journal.pone.0186177 29020103 PMC5636138

[ar70073-bib-0013] Barszcz, K. , Polguj, M. , Klećkowska‐Nawrot, J. , Goździewska‐Harłajczuk, K. , Olbrych, K. , & Czopowicz, M. (2020). Morphometry and topography of the coronary ostia in the European bison. Folia Morphologica, 79, 105–112. 10.5603/FM.a2019.0041 30973638

[ar70073-bib-0014] Biasi, C. , Borelli, V. , Benedicto, H. G. , Pereira, M. R. , Favaron, P. O. , & Bombonato, P. P. (2012). Análise comparativa entre a vascularização ventricular e do nó sinoatrial em gatos. Pesquisa Veterinária Brasileira, 32(1), 78–82. 10.1590/S0100-736X2012000100013

[ar70073-bib-0015] Biasi, C. , Borelli, V. , Prazeres, R. F. , Favaron, P. O. , Pavanello Junior, V. , Aloia, T. P. A. , & Bombonato, P. P. (2013). Análise comparativa entre a vascularização arterial ventricular e a do nó sinoatrial em corações de cães. Pesquisa Veterinária Brasileira, 33(1), 111–114. 10.1590/S0100-736X2013000100020

[ar70073-bib-0016] Borelli, V. (2014). Contribuição ao estudo da vascularização arterial do coração de gatos (Felis domestica–Línnaeus 1758). Journal of the Health Sciences Institute, 32(3), 299–303.

[ar70073-bib-0017] Buchanan, J. W. (1990). Pulmonic stenosis caused by single coronary artery in dogs: Four cases (1965–1984). Journal of the American Veterinary Medical Association, 196(1), 115–120. 10.2460/javma.1990.196.01.115 2295544

[ar70073-bib-0018] Büll, M. L. , & Martins, M. R. F. B. (2002). Study of the arterial coronary circulation in the dog (Canis familiaris). Revista Chilena de Anatomia, 20(2), 117–123. 10.4067/S0716-98682002000200001

[ar70073-bib-0019] Chiarello, A. , Santos, P. , Moraes‐Barros, N. , & Miranda, F. (2022). *Bradypus torquatus*. The IUCN Red List of Threatened Species 2022: e.T3036A210442361. Accessed on 08 October 2023.

[ar70073-bib-0020] Choy, J. S. , & Kassab, G. K. (2008). Scaling of myocardial mass to flow and morphometry of coronary arteries. Journal of Applied Physiology, 104, 1281–1286. 10.1152/japplphysiol.01261.2007 18323461 PMC2629558

[ar70073-bib-0021] Clerici, G. P. , Rosa, P. S. , & Costa, F. R. (2018). Description of digging behavior in armadillos *Dasypus novemcinctus* (Xenarthra: Dasypodidae). Mastozoología Neotropical, 25(2), 283–291.

[ar70073-bib-0022] Cliffe, R. N. , Scantlebury, D. M. , Kennedy, S. J. , Avey‐Arroyo, J. , Mindich, D. , & Wilson, R. P. (2018). The metabolic response of the *Bradypus* sloth to temperature. PeerJ, 6, e5600. 10.7717/peerj.5600 30258712 PMC6151113

[ar70073-bib-0023] Correia‐Oliveira, M. , Hernandez, J. M. F. , & Abidu‐Figueiredo, M. (2013). Morfometria cardíaca e distribuição das artérias coronárias em bovinos mestiços. Biotemas, 26(2), 199–207. 10.5007/2175-7925.2013v26n2p199

[ar70073-bib-0024] Correia‐Oliveira, M. , Moraes, S. O. S. , Gomes, M. S. , Palhano, H. B. , & Abidu‐Figueiredo, M. (2014). Dominância entre as artérias coronárias em bovinos mestiços. Revista Brasileira De Ciência Veterinária, 21, 82–85. 10.4322/rbcv.2014.027

[ar70073-bib-0025] Correia‐Oliveira, M. , Oliveira, I. M. S. , Roza, M. S. , & Abidu‐Figueiredo, M. (2014). Morfometria cardíaca e distribuição das artérias coronárias em coelhos Nova Zelândia (Oryctolagus cunniculus). Revista Brasileira De Medicina Veterinaria, 36, 159–166.

[ar70073-bib-0026] Cruvinel, C. A. T. , Cruvinel, T. M. A. , Aires, L. P. N. , Rodrigues, R. F. , & Melo, A. P. F. (2019). Characterization of coronary arteries in Giant anteater (Myrmecophaga tridactyla: Myrmecophagidae). Arquivo Brasileiro de Medicina Veterinária e Zootecnia, 71(2), 545–552. 10.1590/1678-4162-10112

[ar70073-bib-0027] Cruz, T. L. , Marçal, A. V. , Bombonato, P. P. , Benedicto, H. G. , Carneiro, F. O. , Severino, R. S. , Smrreaux, P. G. , & Blazquez, F. J. H. (2007). Pontes de miocárdio em ovinos da raça Ideal: Frequência e largura. Ciência Animal Brasileira, 8(2), 307–312. 10.5216/cab.v8i2.1355

[ar70073-bib-0028] Delsuc, F. , Ctzeflis, F. M. , Stanhope, M. J. , & Douzery, E. J. P. (2001). The evolution of armadillos, anteaters and sloths depicted by nuclear and mitochondrial phylogenies: implications for the status of the enigmatic fossil Eurotamandua. Proceedings of the Royal Society B: Biological Sciences, 268(1476), 1605–1615. 10.1098/rspb.2001.1702 PMC1088784 11487408

[ar70073-bib-0029] Delsuc, F. , Scally, M. , Madsen, O. , Stanhope, M. J. , de Jong, W. W. , Catzeflis, F. M. , & Douzery, E. J. P. (2002). Molecular phylogeny of living xenarthrans and the impact of character and taxon sampling on the placental tree rooting. Molecular Biology and Evolution, 19(10), 1656–1671. 10.1093/oxfordjournals.molbev.a003989 12270893

[ar70073-bib-0030] Donald, D. E. , & Essex, H. E. (1954). Pressure studies after inactivation of the major portion of the canine right ventricle. American Journal of Physiology, 176, 155–161. 10.1152/ajplegacy.1953.176.1.155 13124512

[ar70073-bib-0031] Dowd, D. A. (1969). The coronary vessels and conducting system in the heart of monotremes. Acta Anatomica, 74, 547–573. 10.1159/000143418 5375648

[ar70073-bib-0032] Dowd, D. A. (1974). The coronary vessels in the heart of a marsupial (*Trichosurus vulpecula*). American Journal of Anatomy, 140(1), 47–56. 10.1002/aja.1001400104 4596335

[ar70073-bib-0033] Durán, A. C. , Fernández, M. C. , Fernández, B. , Fernández‐Gallego, T. , Arqué, J. M. , & Sans‐Coma, V. (2007). Number of coronary ostia in Syrian hamsters (*Mesocricetus auratus*) with normal and anomalous coronary arteries. Anatomia, Histologia, Embryologia, 36(6), 460–465. 10.1111/j.1439-0264.2007.00788.x 18021357

[ar70073-bib-0034] Engelmann, G. F. (1985). The phylogeny of the Xenarthra. In G. G. Montgomery (Ed.), The evolution and ecology of armadillos, sloths, and vermilinguas (pp. 51–64). Smithsonian Institution Press.

[ar70073-bib-0035] Engen, L. R. (2006). Dinâmica do sistema cardiovascular. In Dukes‐Fisiologia dos Animais Domésticos (12th ed.). Guanabara Koogan.

[ar70073-bib-0036] Evans, H. E. , & de Lahunta, A. (2013). Miller's anatomy of the dog (4th ed.). Elsevier.

[ar70073-bib-0037] Farias, É. L. P. , Sousa, R. S. , & Gomes, F. G. F. L. R. (2016). Ocorrência e Morfometria de Pontes de Miocárdio em Cães. Archives of Veterinary Science, 21(3), 82–91. 10.5380/avs.v21i4.46093

[ar70073-bib-0038] Gaggini, T. S. , Zangeronimo, M. G. , Birck, A. J. , & Filadelpho, A. L. (2011). Estudo anatômico das pontes de miocárdio em duas linhagens de suínos comerciais. Revista Científica Eletrônica de Medicina Veterinária, 17, 1–11.

[ar70073-bib-0039] Getty, R. (1986). Anatomia dos Animais Domésticos (5th ed.). Guanabara Koogan.

[ar70073-bib-0040] Glass, B. P. (1985). History of classification and nomenclature in Xenarthra (Edentata). In G. G. Montgomery (Ed.), The evolution and ecology of armadillos, sloths and vermilinguas (pp. 1–4). Smithsonian Institution Press.

[ar70073-bib-0041] Guyton, A. C. , & Hall, J. E. (1997). Tratado de Fisiologia Médica (9th ed.). Guanabara Koogan.

[ar70073-bib-0042] International Committee on Veterinary Gross Anatomical Nomenclature . (2017). Nomina Anatômica Veterinária (6th ed.). Editorial Committee.

[ar70073-bib-0043] Jensen, B. , Moorman, A. F. M. , & Wang, T. (2014). Structure and function of the hearts of lizards and snakes. Biological Review, 89, 302–336. 10.1111/brv.12056 23998743

[ar70073-bib-0044] Kim, S. Y. , Seo, J. B. , Do, K. H. , Heo, J. N. , Lee, J. S. , Song, J. W. , Choe, Y. H. , Kim, T. H. , Yong, H. S. , Choi, S. I. , Song, K. S. , & Lim, T. H. (2006). Coronary artery anomalies: classification and ECG‐gated multi‐detector row CT findings with angiographic correlation. Radiographics, 26(2), 317–334. 10.1148/rg.262055068 16549600

[ar70073-bib-0045] Kosiński, A. , & Grzybiak, M. (2001). Myocardial bridges in the human heart: morphological aspects. Folia Morphologica, 60(1), 65–68. 11234701

[ar70073-bib-0046] Loukas, M. , Curry, B. , Bowers, M. , Louis, R. G. , Bartczak, A. , Kiedrowski, M. , Kamionek, M. , Fudalej, M. , & Wagner, T. (2006). The relationship of myocardial bridges to coronary artery dominance in the adult human heart. Journal of Anatomy, 209(1), 43–50. 10.1111/j.1469-7580.2006.00590.x 16822268 PMC2100301

[ar70073-bib-0047] Machado, M. R. F. , Borges, E. M. , Oliveira, F. S. , Filippini‐Tomazini, M. , de Melo, A. P. F. , & Duarte, J. M. B. (2002). Intramyocardial course of the coronary arteries in the marsh deer (*Blastocerus dichotomus*). Brazilian Journal of Veterinary Research and Animal Science, 39(6), 285–287. 10.1590/S1413-95962002000600002

[ar70073-bib-0048] Mackinnon, M. R. , & Heatwole, H. (1981). Comparative cardiac anatomy of the reptilia. IV. The coronary arterial circulation. Journal of Morphology, 170, 1–27. 10.1002/jmor.1051700102 7288884

[ar70073-bib-0049] Madsen, O. , Scally, M. , Douady, C. J. , Kao, D. J. , DeBry, R. W. , Adkins, R. , Amrine, H. M. , Stanhope, M. J. , Jong, W. W. , & Springer, M. S. (2001). Parallel adaptive radiations in two major clades of placental mammals. Nature, 409, 610–614. 10.1038/35054544 11214318

[ar70073-bib-0050] Maia, O. B. (2002). Maternal behavior of two captive giant anteaters Myrmecophaga tridactyla Linnaeus, 1758. Revista de Etología, 4(1), 41–47.

[ar70073-bib-0051] Marshall, S. K. , Superina, M. , Spainhower, K. B. , & Butcher, M. T. (2021). Forelimb myology of armadillos (Xenarthra: Cingulata, Chlamyphoridae): Anatomical correlates with fossorial ability. Journal of Anatomy, 238(3), 551–575. 10.1111/joa.13326 33111984 PMC7855086

[ar70073-bib-0052] McKenna, M. C. , & Bell, S. K. (1997). Classification of mammals above the species level. Columbia University Press.

[ar70073-bib-0053] Medri, I. M. , Mourão, G. , & Rodrigues, F. H. G. (2011). Ordem Pilosa. In N. R. Reis , L. Peracchi , W. A. Pedro , & A. P. Lima (Eds.), Mamíferos do Brasil (2nd ed., pp. 91–101). Eduel.

[ar70073-bib-0054] Melo, F. A. C. , Lima, E. M. M. , Santana, M. I. S. , & Benedicto, H. G. (2010). Pontes de miocárdio em ovinos da raça Santa Inês. Ars Veterinaria, 26(1), 43–46.

[ar70073-bib-0055] Miranda, F. , Bertassoni, A. , & Abba, A. M. (2014). Myrmecophaga tridactyla. The IUCN Red List of Threatened Species 2014: e.T14224A47441961. 10.2305/IUCN.UK.2014-1.RLTS.T14224A47441961.en

[ar70073-bib-0056] Miranda, F. R. , Garbino, G. S. T. , Machado, F. A. , Perini, F. A. , Santos, F. R. , & Casali, D. M. (2023). Taxonomic revision of maned sloths, subgenus Bradypus (Scaeopus), Pilosa, Bradypodidae, with revalidation of Bradypus crinitus Gray, 1850. Journal of Mammalogy, 104(1), 86–103. 10.1093/jmammal/gyac059

[ar70073-bib-0057] Monfared, A. L. , Moosavi, S. , & Bazdar, A. (2013). The macroanatomy of coronary arteries in the Iranian native cats. Global Veterinaria, 10(4), 413–416.

[ar70073-bib-0058] Moore, R. A. (1930). The coronary arteries of the dog. American Heart Journal, 5(6), 743–749. 10.1016/S0002-8703(30)90089-0

[ar70073-bib-0059] Moura Junior, P. C. , Vieira, T. H. M. , Vieira, S. R. C. , Sobreiro, D. , Ruiz, C. R. , Gabriela, C. , Wafae, G. C. , Silva, N. C. , & Wafae, N. (2008). Estudo anatômico das artérias coronárias de suínos Landrace. Pesquisa Veterinária Brasileira, 28, 103–107. 10.1590/S0100-736X2008000200002

[ar70073-bib-0060] Murphy, W. J. , Eizirik, E. , O'Brien, S. J. , Madsen, O. , Scally, M. , Douady, C. J. , Teeling, E. , Ryder, O. A. , Stanhope, M. J. , De Jong, W. W. , & Springer, M. S. (2001). Resolution of the early placental mammal radiation using Bayesian phylogenetics. Science, 294(5550), 2348–2351. 10.1126/science.1067179 11743200

[ar70073-bib-0061] Nocetti, L. M. , Bombonato, P. P. , Santana, M. I. S. , Carneiro e Silva, F. O. , & Severino, R. S. (2002). Pontes de miocárdio em corações de javali. Brazilian Journal of Veterinary Research and Animal Science, 39(2), 66–73. 10.1590/S1413-95962002000200002

[ar70073-bib-0062] Noestelthaller, A. , Probst, A. , & König, H. E. (2007). Branching patterns of the left main coronary artery in the dog demonstrated by the use of corrosion casting technique. Anatomia, Histologia, Embryologia, 36, 33–37. 10.1111/j.1439-0264.2006.00711.x 17266665

[ar70073-bib-0063] Olabu, B. O. , Saidi, H. S. , Hassanali, J. , & Ogeng'o, J. A. (2007). Prevalence and distribution of the third coronary artery in Kenyans. International Journal of Morphology, 25, 851–854. 10.4067/S0717-95022007000400027

[ar70073-bib-0064] Oliveira, C. L. , Dornelas, D. , Carvalho, M. O. , Wafae, G. , David, G. S. , Araújo, S. , Silva, N. C. , Ruiz, C. R. , & Wafae, N. (2010). Anatomical study on coronary arteries in dogs. European Journal of Anatomy, 14, 1–4.

[ar70073-bib-0065] Oliveira, C. L. S. , David, G. S. , Carvalho, M. O. , Dornelas, D. , Araújo, S. , Silva, N. C. , Ruiz, C. R. , Fernandes, J. R. , & Wafae, N. (2011). Anatomical indicators of dominance between the coronary arteries of dogs. International Journal of Morphology, 29(3), 845–849. 10.4067/S0717-95022011000300030

[ar70073-bib-0066] Pereira, V. P. , Prates, B. M. , Seyfert, C. E. , & de Morais‐Pinto, L. (2022). Morphological importance of coronary ostia in sheep and swine. Anatomia, Histologia, Embryologia, 51, 339–346. 10.1111/ahe.12793 35165926

[ar70073-bib-0067] Pereira, V. P. , Seyfert, C. E. , Santos, J. M. L. , & de Morais‐Pinto, L. (2022). Morphological importance of coronary ostia in equine. Anatomia, Histologia, Embryologia, 51, 658–665. 10.1111/ahe.12844 35894158

[ar70073-bib-0068] Pinheiro, G. S. , Branco, É. , Pereira, L. C. , & Lima, A. R. (2014). Morfologia, topografia e irrigação do coração do *Tamandua tetradactyla* . Arquivo Brasileiro de Medicina Veterinária e Zootecnia, 66(4), 1105–1111. 10.1590/1678-6844

[ar70073-bib-0069] Pough, F. H. , Heiser, J. B. , & McFarland, W. N. (1993). A Vida dos Vertebrados. Atheneu.

[ar70073-bib-0070] Rade, W. , Felipe Pereira, W. , & Ozanan Carneiro e Silva, F. (2006). Origem, trajeto, distribuição e ramificações ventriculares da artéria coronária direita do macaco prego (Cebus apella). Bioscience Journal, 22, 133–137.

[ar70073-bib-0071] Rai, G. , Khanna, S. , & Singh, R. (2020). Myocardial bridging in the course of coronary arteries and its clinical significance. Asian Journal of Medical Sciences, 11(6), 58–62. 10.3126/ajms.v11i6.29989

[ar70073-bib-0072] Ribeiro, A. L. C. , Severino, R. S. , Guerra, R. R. , Favaron, P. O. , Tommasi Junior, H. L. P. , Ricci, R. E. G. , Franciolli, A. L. R. , Facciotti, P. R. , & Bombonato, P. P. (2009). Biometria de Pontes de Miocárdio em Muares (Equus caballus × Equus asinus—Linnaeus 1758). Biotemas, 22(3), 177–184. 10.5007/2175-7925.2009v22n3p177

[ar70073-bib-0073] Ribeiro, L. A. , Borges, T. R. J. , Santos, L. A. , Silva, F. O. C. , Santana, A. A. , Silva, L. K. , Rabelo, J. C. A. , & Ribeiro, C. J. (2014). Descrição das artérias coronárias de javali (Sus scrofa Linnaeus, 1758). In Laboratório de Pesquisa em Animais Silvestres (Org.). *Anais do VII Encontro sobre Animais Selvagens e II Simpósio sobre Medicina e Conservação da Fauna do Cerrado*. Universidade Federal de Uberlândia. Retrieved from http://www.pet.famev.ufu.br/sites/pet.famev.ufu.br/files/CADERNO%20DE%20RESUMOS%20atualizado.pdf

[ar70073-bib-0074] Rodrigues, M. R. (1997). *Morfologia do coração do gato doméstico (Felis catus): vascularização arterial e região juncional atrioventricular* [Dissertação de Mestrado em Biologia Humana e Experimental, Universidade do Estado do Rio de Janeiro].

[ar70073-bib-0075] Sans‐Coma, V. , Arqué, J. M. , Durán, A. C. , Cardo, M. , Fernández, B. , & Franco, D. (1993). The coronary arteries of the Syrian hamster, *Mesocricetus auratus* (Waterhouse 1839). Annals of Anatomy, 175(1), 53–57. 10.1016/s0940-9602(11)80239-6 8465975

[ar70073-bib-0076] Santos, N. R. L. , Benetti, E. J. , Oliveira, K. M. , Medeiros, M. V. M. , & Simões, K. (2020). Heart structure and coronary blood supply of the Giant anteater (*Myrmecophaga tridactyla*). Anatomia, Histologia, Embryologia, 50, 15–22. 10.1111/ahe.12594 32686854

[ar70073-bib-0077] Santos, T. F. A. , Rodrigues Godinho, A. B. F. , Almeida, S. M. , Leandro, H. J. , & Quirino, C. R. (2022). Estudo morfológico de pontes de miocárdio em cães. Pubvet, 15(10), 1–7. 10.31533/pubvet.v15n10a950.1-7

[ar70073-bib-0078] Scansen, B. A. (2017). Coronary artery anomalies in animals. Veterinary Sciences, 4, 20. 10.3390/vetsci4020020 29056679 PMC5606599

[ar70073-bib-0079] Schiller, H. J. (1957). Das Herz des Lowen (Felis leo L.): Beitrag zur Anatomie des Carnivorenherzens. Gegenbaurs Morphologisches Jahrbuch, 100, 163–184.

[ar70073-bib-0080] Schlesinger, M. J. , Zoll, P. M. , & Wessler, S. (1949). The conus artery: A third coronary artery. American Heart Journal, 38, 823–836. 10.1016/0002-8703(49)90884-4 15395916

[ar70073-bib-0081] Schummer, A. , Wilkens, H. , Vollmerhaus, B. , & Habermehl, K.‐H. (1981). The circulatory system, the skin and the cutaneous organs of the domestic mammals. Verlag Paul Parey. 10.1007/978-1-4899-7102-9

[ar70073-bib-0082] Severino, R. S. , & Bombonato, P. P. (1992). Ocorrência de pontes de miocárdio em bovinos das raças Gir, Guzerá, Indubrasil e Nelore. Brazilian Journal of Veterinary Research and Animal Science, 29(1), 15–30. 10.11606/issn.1678-4456.bjvras.1992.51948

[ar70073-bib-0083] Severino, R. S. , Carneiro e Silva, F. O. , Santos, A. L. Q. , Drummond, S. S. , Bombonato, P. P. , Duran, F. P. , & Marçal, A. V. (1997). Pontes de miocárdio em bovinos azebuados. Brazilian Journal of Veterinary Research and Animal Science, 34(5), 288–291.

[ar70073-bib-0084] Shriki, J. E. , Shinbane, J. S. , Rashid, M. A. , Hindoyan, A. , Withey, J. G. , DeFrance, A. , Cunningham, M. , Oliveira, G. R. , Warren, B. H. , & Wilcox, A. (2012). Identifying, characterizing, and classifying congenital anomalies of the coronary arteries. Radiographics, 32(2), 453–468. 10.1148/rg.322115097 22411942

[ar70073-bib-0085] Singh, B. (2018). Dyce, sack and Wensing's textbook of veterinary anatomy (5th ed.). Elsevier.

[ar70073-bib-0086] Springer, M. S. , Murphy, W. J. , Eizirik, E. , & O'Brien, S. J. (2003). Placental mammal diversification and the cretaceous‐tertiary boundary. Proceedings of the National Academy of Sciences of the United States of America, 100(3), 1056–1061. 10.1073/pnas.0334222100 12552136 PMC298725

[ar70073-bib-0087] Srour, H. A. (2011). *Origin and ventricular ramifications of the left coronary artery in capuchin monkey (Cebus apella)* [Dissertação de mestrado, Universidade Federal de Uberlândia]. Repositório Institucional ‐ Universidade Federal de Uberlândia. 10.14393/ufu.di.2011.47

[ar70073-bib-0088] Viotto‐Souza, W. , de Souza Junior, P. , de Carvalho, A. D. , Abidu‐Figueiredo, M. , & Santos, A. L. Q. (2017). Coronary irrigation in Puma concolor (Carnivora: Felidae). International Journal of Morphology, 35(3), 925–930. 10.4067/S0717-95022017000300021

[ar70073-bib-0089] Viotto‐Souza, W. , Santos, A. L. Q. , Abidu‐Figueiredo, M. , Kasper, C. B. , Carvalho, A. D. , & Souza‐Junior, P. (2023). Coronary anatomy in neotropical carnivores: A comparative analysis. Anatomical Record, 307(6), 2149–2161. 10.1002/ar.25357 38058234

[ar70073-bib-0090] Vizcaíno, S. F. , & Milne, N. (2002). Structure and function in armadillo limbs (Mammalia: Xenarthra: Dasypodidae). Journal of Zoology, 257(2), 117–127. 10.1017/S0952836902000717

[ar70073-bib-0091] Vizcaíno, S. F. , & Scillato‐Yané, G. J. (1995). An Eocene tardigrade (Mammalia, Xenarthra) from Seymour Island, West Antarctica. Antarctic Science, 7(4), 407–408. 10.1017/S0954102095000563

[ar70073-bib-0092] Yoldas, A. , Ozmen, E. , & Ozdemir, V. (2010). Macroscopic description of the coronary arteries in Swiss albino mice (*Mus musculus*). Journal of the South African Veterinary Association, 81(4), 247–252. 10.4102/jsava.v81i4.156 21526741

[ar70073-bib-0093] Yuan, G. , Ma, J. , Ye, W. , Bai, Z. , & Wang, J. (2009). Macroanatomy of coronary arteries in Bactrian camel (*Camelus bactrianus*). Veterinary Research Communications, 33(4), 367–377. 10.1007/s11259-008-9185-0 19011985

[ar70073-bib-0094] Zaniboni, M. S. , Viotto‐Souza, W. , Samora, D. G. A. , Bernardes, F. C. S. , Santos, A. L. Q. , Carvalho, A. D. , & Souza‐Junior, P. (2021). Angioarchitecture of the coronary arteries in Mazama gouazoubira (G. Fischer, 1814). Acta Veterinaria Brasilica, 15, 297–303. 10.21708/avb.2021.15.4.10007

[ar70073-bib-0095] Zhang, J. , Yu, S.‐Y. , Xie, M.‐R. , Chen, Y.‐Q. , Shang, J.‐K. , & Zhang, H.‐L. (2008). Anatomy of the coronary arteries in *Panthera pardus* and *Arctonyx collaris* . Chinese Journal of Zoology, 43, 144–149.

